# Rigid Polyurethane Foams’ Development and Optimization from Polyols Based on Depolymerized Suberin and Tall Oil Fatty Acids

**DOI:** 10.3390/polym16070942

**Published:** 2024-03-29

**Authors:** Aiga Ivdre, Mikelis Kirpluks, Arnis Abolins, Laima Vevere, Beatrise Sture, Aigars Paze, Daniela Godina, Janis Rizikovs, Ugis Cabulis

**Affiliations:** Latvian State Institute of Wood Chemistry, 27 Dzerbenes Str., LV-1006 Riga, Latvia; aiga.ivdre@kki.lv (A.I.); mikelis.kirpluks@kki.lv (M.K.); arnis.abolins@kki.lv (A.A.); laima.vevere@kki.lv (L.V.); beatrise.sture@kki.lv (B.S.); aigars.paze@kki.lv (A.P.); daniela.godina@kki.lv (D.G.); janis.rizikovs@kki.lv (J.R.)

**Keywords:** suberin, tall oil, bio-based polyurethanes, rigid polyurethane foam

## Abstract

The utilization of polyols derived from renewable sources presents an opportunity to enhance the sustainability of rigid polyurethane (PUR) foams, thereby contributing to the advancement of a circular bioeconomy. This study explores the development of PUR rigid foams exclusively using polyols sourced from second-generation renewable biomass feedstocks, specifically depolymerized birch bark suberin (suberinic acids) and tall oil fatty acids. The polyols achieved a total renewable material content as high as 74%, with a suberinic acid content of 37%. Response surface modeling was employed to determine the optimal bio-polyol, blowing agents, and catalyst content, hence, optimizing the bio-based foam formulations. In addition, response surface modeling was applied to rigid PUR foam formulations based on commercially available petroleum-based polyols for comparison. The results, including apparent density (~40–44 kg/m^3^), closed cell content (~95%), compression strength (>0.2 MPa, parallel to the foaming direction), and thermal conductivity (~0.019 W/(m·K)), demonstrated that the suberinic acids-based rigid PUR foam exhibited competitive qualities in comparison to petroleum-based polyols. Remarkably, the bio-based rigid PUR foams comprised up to 29% renewable materials. These findings highlight the potential of suberinic acid-tall oil polyols as effective candidates for developing rigid PUR foams, offering promising solutions for sustainable insulation applications.

## 1. Introduction

Global warming, climate change, and the finite nature of petrochemical resources have brought sustainability to the forefront of global concerns. In response, the concept of sustainability has evolved into a multidimensional framework encompassing environmental, economic, and social aspects. Within this framework, the circular bio-economy has emerged as a pivotal approach to enhancing sustainability by optimizing resource use and minimizing waste generation [1,2].

At the heart of the circular bio-economy lies the imperative to transition away from fossil-derived materials towards renewable alternatives. This shift entails a comprehensive re-evaluation of material sourcing and production processes across various industries. Plastics, ubiquitous in modern society, epitomize this challenge. Historically, plastics have been predominantly derived from fossil fuels, including coal, natural gas, and crude oil [3]. However, the environmental impacts associated with fossil fuel extraction and the accumulation of plastic waste in terrestrial and marine ecosystems have spurred urgent calls for alternative solutions.

Polyurethane (PUR), a versatile polymer with a myriad of applications ranging from construction materials to consumer products, stands as a prime example of the need for sustainable alternatives in the plastics sector. Rigid PUR foams, utilized extensively for thermal insulation in buildings and appliances, represent a significant market segment [4]. Yet, the production of PUR traditionally relies on polyols derived from fossil fuels, underscoring the critical importance of exploring bio-based alternatives.

The evolution of bio-based polyols mirrors the broader trajectory of sustainability efforts within the circular bio-economy. Initially, bio-based polyols predominantly relied on first-generation biomass feedstocks, such as edible oils and crops. However, concerns regarding the competition with food sources and land use conflicts have prompted a shift towards second-generation feedstocks. These include waste materials, wood biomass derivatives, and inedible oils, offering the potential to decouple polyol production from food-related markets [5,6,7]. Among the promising sources of bio-based polyols, inedible oils have garnered significant attention. These oils, derived from sources such as algae [8,9], microbial fermentation, and non-food plant sources [10,11], offer a sustainable alternative to conventional polyol feedstocks [12]. By utilizing inedible oils, the circular bio-economy seeks to minimize competition with food production while simultaneously reducing greenhouse gas emissions associated with traditional fossil fuel-based processes [13]. The utilization of waste biomass streams further exemplifies the circular bio-economy’s commitment to maximizing resource efficiency. Two second-generation biomass feedstocks, used in this study, will be described in more detail—tall oil and suberin.

Tall oil, a byproduct of the Kraft pulping process of pine wood, is an inedible oil considered lignocellulosic plant biomass waste. It primarily comprises rosin acids and fatty acids. Notably, tall oil fatty acids (TOFA) possess two reactive sites in their molecules—a carboxylic group and one to three double bonds—making them suitable for both esterification and epoxidation reactions. This characteristic allows for the synthesis of polyols from TOFA or epoxidized TOFA (ETOFA) for PUR production [7,14].

Birch bark, a byproduct of various industrial processes, represents a valuable source of biomass for energy production [15]. Beyond its calorific value, birch bark contains compounds with potential industrial applications, including suberin and suberinic acids (SA) obtained from it [16,17,18], as well as triterpene-rich extracts like betulin and lupeol [15]. Through innovative extraction and processing techniques, these compounds can be transformed into high-value products, contributing to the economic viability of biomass utilization [19]. After triterpene extraction, the remaining biomass can still be used for the production of other high-added-value products from suberin [20,21,22], which can be converted to SA by chemical treatment to break down its components into smaller, more valuable compounds. This can be achieved through various processes, such as hydrolysis or alkaline treatment, to extract or modify specific constituents—SA. SA obtained in the water-alkaline depolymerization processes of the outer bark of birch, have been studied for their potential as particle board [23] and plywood adhesives [24]. Due to the large number of aliphatic and aromatic hydroxyl groups, SA can be used as a polyol in PUR synthesis. ω-hydroxy fatty acids, prevalent in the outer bark of certain birch species, serve as key building blocks for polymer synthesis [21]. If SA are obtained in an ethanol alkaline depolymerization process they can be used to synthesize polyols. The incorporation of SA polyols can influence the curing process, foam formation, and the overall structure and properties of the PUR material. SA-modified polyols can be blended with other polyols to achieve desired properties in the final product [21,25,26]. The potential for SA-based rigid PUR foam production as thermal insulation material has been demonstrated. However, a potential obstacle to the commercial application of SA-based polyols could be their high viscosity [27].

In conclusion, this represents a significant advancement in the quest for sustainable alternatives to fossil-derived polyols, paving the way for the integration of renewable biomass into the mainstream polymer production processes. The transition towards sustainability within the plastics industry epitomizes the broader challenges and opportunities inherent in the circular bio-economy. By reimagining material sourcing, production processes, and waste management strategies, stakeholders across various sectors can work towards a more resilient and regenerative economic model. The case of bio-based polyols derived from inedible oils and waste biomass exemplifies this paradigm shift, offering a glimpse into the potential of biomimicry and biorefinery concepts to drive innovation and sustainability in the 21st century.

In our study, we aimed to address the challenge posed by the high viscosity of SA-based polyols by modifying the polyol synthesis methodology. To accomplish this goal, SA-based polyols modified with TOFA were synthesized for the first time, employing various molar ratios and types of multifunctional alcohols as transesterification reagents. Additionally, polyols with higher functionality were synthesized from SA and ETOFA. From the resulting polyols, a lower functionality and a higher functionality polyol were selected to construct a multi-variable response surface model (MRSM). Also, MRSM was developed from conventional polyols. These models facilitated the investigation of multiple factors affecting the desired properties of rigid PUR foams. Subsequently, the optimized rigid PUR foams were evaluated for thermal insulation characteristics and compared with foams from conventional polyols.

## 2. Materials and Methods

### 2.1. Materials for Polyol Synthesis

The materials used to obtain SA-based polyols are summarized in Table 1.

### 2.2. Synthesis of SA-Based Polyols

The SA-based polyol synthesis was carried out in a four-necked round bottom flask. The flask was immersed in the oil bath equipped with a mechanical stirrer, a Liebig condenser, a thermocouple, and a gas inlet tube. The required amount of SA fraction (SA-C or SA-E) and TOFA (25 wt.%, 50 wt.% and 75 wt.%) or ETOFA (25 wt.%, 50 wt.% and 75 wt.%) in respect to SA fraction were introduced into the flask. After that, the required amount of polyfunctional alcohol and catalyst (0.5% of synthesis mass) was added to the reaction medium. When all reagents were added, the flask was immersed into the oil bath and stirred with anchor type mixer at 60 rpm. The inert gas (20 mL/min) was applied at the bottom of the reactor through a capillary inlet. As the reaction medium reached approximately 70 °C, it began to homogenize, prompting an increase in stirring to 500 rpm. The temperature was then raised to the predetermined synthesis temperature and maintained until the end of the synthesis process. The selection of the synthesis temperature was based on our previous research [27]. For synthesis involving BD, EG, DEG, and TMP, the temperature was set at 185 ± 5 °C, while for DEOA and TEOA, it was maintained at 145 ± 5 °C and 175 ± 5 °C, respectively.

The molar ratios between reactants for polyol synthesis were calculated using Equation (1):

n_(PA)_ = n_(CG)_, (1)

where: n_(PA)_—molar amount of the polyfunctional alcohol used for synthesis (BD, EG, DEG, DEOA, TEOA or TMP), mol; n_(CG)_—molar amount of the raw material combined carboxylic groups (acid value), mol.

When the SA fraction with increased epoxy group content and ETOFA were used, the required molar amount of polyfunctional alcohol for polyol synthesis was determined using Equation (2):

n_(PA)_ = n_(CG)_ + n_(OG)_, (2)

where: n_(PA)_—molar amount of the polyfunctional alcohol used for oxirane ring-opening and esterification reaction (TMP or TEOA), in mol; n_(CG)_ is the molar amount of the raw material combined carboxylic groups, in mol and n_(OG)_ is the molar amount of the raw material combined oxirane groups, in mol.

### 2.3. Characterization of the Synthesized SA-Based Polyols

After synthesis, the obtained bio-polyol was analyzed for the OH value and apparent viscosity to assess suitability for rigid PUR foam development.

The titrimetric analyses were performed using TitroLine^®^ 7000 automatic titrator (Xylem Analytics Germany GmbH, Weilheim, Germany). The characteristics of polyols were determined according to the methods presented in Table 2.

The rheological tests were performed on an Anton Paar Rheometer MCR 92 (Anton Paar, Graz, Austria). The experiments were carried out by using a cone-plate system with a gap of 48 μm. Shear rate ramps ranging from 1 s^−1^ to 100 s^−1^ were performed. The measurements were carried out at a constant temperature of 25 °C, which was maintained by the temperature hood. Using Karl Fisher titration, the moisture content was measured using the Denver Instrument Model 275 KF automatic titrator (Denver Instrument, Bohemia, NY, USA).

### 2.4. Materials for Rigid PUR Foam Production

Two series of rigid PUR foams were obtained based on two types of polyols (bio-based polyols and commercially used polyols from petroleum feedstock):

1. LP series—rigid PUR foams from commonly used polyether polyols purchased from BASF, Germany (Lupranol 3300 and Lupranol 3422);
2. SP series—rigid PUR foams from synthesized SA-based polyols (SA-C_TOFA(50:50)/TMP and SA-E_ETOFA(50:50)/TMP);

SA-based polyols for the SP series were chosen as the two most suitable polyols from synthesized polyols characterized further in Section 3.1. Lupranol 3300 (OH = 400 mgKOH/g) and SA-C_TOFA(50:50)/TMP were defined as low functionality polyols (LF polyols) whereas SA-E_ETOFA(50:50)/TMP and Lupranol 3422 (OH = 490 mgKOH/g) were defined as high functionality polyols.

In addition to polyols, the materials listed below were utilized as bought for the polyol component: catalysts PC CAT TKA 30^®^ and PC CAT NP10^®^ (Air Products and Chemicals Inc., Allentown, PA, USA); flame retardant tris(1-chloro-2-propyl)phosphate (TCPP) (Albermarle, Charlotte, NC, USA); surfactant Niax Silicone L-6915 (Momentive Performance Materials Inc., Leverkusen, Germany); physical blowing agent Opteon™ 1100 (Chemours, Wilmington, DE, USA) and chemical blowing agent distilled water. Desmodur 44 V20 L (pMDI) (Covestro, Augusta, GA, USA) was employed as the isocyanate component. It is a product based on 4,4′-diphenylmethane diisocyanate that is solvent-free and has high-functionality oligomers. The NCO group content is 31.5 wt.%, and the functionality of pMDI ranges from 2.8 to 2.9 on average.

### 2.5. Rigid PUR Foam Formulations for Multi-Variable Response Surface Modeling

The MRSM method was chosen as the most convenient way to investigate the influence of several factors on the desired properties of the rigid PUR foams. An experimental matrix was created in Design Expert software (Version V12.0.7.0, Stat-Ease, Inc., Minneapolis, MN, USA) to investigate four different factors’ influence on the rigid PUR foam properties. The influence of polyol ratios, amount of physical blowing agent Opteon™ 1100, chemical blowing reagent water, and catalyst PC CAT NP10^®^ content on the apparent density, closed cell content, foaming start time, foaming rise time, shrinkage, renewable material content in the foam, and dimensional stability was determined. A response surface study was carried out using the Box–Behnken design type. The factor influence on the rigid PUR properties was approximated by a linear or quadratic polynomial model. Modeling was carried out for both, LP and SP rigid PUR foam series. The PUR formulations with changed factors are depicted in Table 3, whereas Table S1 describes the changed factors and the coded levels of the MRSM.

### 2.6. Optimization of the Formulations of Rigid PUR Foams

A mathematical approach to finding the optimal rigid PUR foam formulation was used to calculate a desirability function *D*(*X*) which reflects the desirable range (*d_i_*) for the different factors (low OH group functionality polyol content; catalyst content, Opteon™1100 content, and water content) and obtained responses (apparent density, closed cell content, foaming start time, foaming rise time, shrinkage after 24 h and renewable material content in the end material). The desirable ranges from the least to most desirable were set from zero to one, respectively. Equation (3) describes the simultaneous objective function, which is a geometric mean of all converted factors and responses, where *n* is the number of factors and responses:

(3) $$D={\left(d_{1}^{r_{1}}\cdot d_{2}^{r_{1}}\cdot \ldots \cdot d_{n}^{r_{n}}\right)}^{\frac{1}{\sum r_{i}}}={\left(\prod_{i=1}^{n}d_{i}^{r_{i}}\right)}^{\frac{1}{\sum r_{i}}}$$

Moreover, a different value of importance was assigned for each factor or response to prioritize the more essential ones. The importance (r_i_) was scaled from 1 to 5, with 1 being the least significant and 5 being the most important. The overall function becomes 0 if the response or factors fall outside their desirability range [33]. The 3D surface plots of the desirability function can be used to explore the function in the factor space.

The optimization parameters for the LP series rigid PUR foams are depicted in Table 4, but for the SP series based rigid PUR foams are depicted in Table 5. The tables depict the parameter optimization, their lower and upper limits, and the importance factors that were attributed to each parameter at optimization.

### 2.7. Rigid PUR Foam Preparation

For MRSM, cup tests were carried out. In summary, 27 formulations were created for each series, and three samples for every formulation were foamed as free-rise foams. That gives 81 samples for each of the series. All ingredients of polyol components according to formulations were weighted and mixed in a plastic cup with a mechanical stirrer for ~2 min at a rate of 2000 rpm, with the exception of the physical blowing agent. Opteon™ 1100 was added as the last ingredient in the polyol component and mixed for ~1 min. Afterward, pMDI was added and mixed for 10 s. The foam was allowed to free-rise in the same cup. Foaming start and rise time, foams’ apparent density, closed cell content, and shrinkage were tested as described further in Section 2.8. In addition, renewable material content and SA content were calculated.

To produce larger samples of optimized rigid PUR foams, the mixing process followed the same steps described above for cup tests. After adding and thoroughly mixing pMDI, the reaction mixture was poured into a larger mold (30 × 30 × 10 cm). Before any additional testing, all of the foam samples were given 24 h to cure at room temperature. Apparent density, closed-cell content, thermal conductivity, compressive strength, compressive modulus, thermal stability, glass transition temperature, and average cell diameter were measured.

### 2.8. Rigid PUR Foam Characterization

The universal foam certification system Foamat was used to evaluate the shrinkage after 24 h as well as the foaming start and rise time.

The gel time, often referred to as the string time was determined by cup test methodology: repeatedly dipping and pulling a glass rod out of the reaction liquid. The gel time was reached when the strings started to emerge. The glass rod was then used to lightly contact the foam’s surface several times until there was no foam adhering to the rod, and then it was recorded as tack-free time. The resultant PUR foams’ apparent density was measured following ISO 845:2006 [34].

Using a pycnometer AccuPyc II 1340, the closed cell content was measured and computed according to ISO 4590:2016 [35].

A FOX 200 was used by TA instruments-Water LLC to test the thermal conductivity coefficient (λ) at an average temperature of 10 °C (cold plate: 0 °C, and hot plate: +20 °C, sample dimensions: 200 × 200 × 30 mm), following the ISO 8301:1991 standard [36].

With a razor blade, thin slices of foam were cut parallel and perpendicular to the direction of the foam rise. The cellular structure of PUR foams was examined under a light microscope Diamond MCXMP500, MICROS Produktions-&Handels GmbH (Sankt Veit an der Glan, Austria), at a 10× magnification. With the use of ImageJ 1.54d software, the collected images were analyzed, and the average cell diameter was determined by ASTM D 3576 [37] in both directions parallel to and perpendicular to the direction in which the foam rose. It was considered that the cell size distribution around the average cell size was normal and that there was no discernible difference in the average cell size from edge to edge or from top to bottom. By dividing the average cell diameter parallel to the foaming direction by the average cell size perpendicular to the foaming direction, geometric anisotropy was calculated.

According to the requirements of the ISO 844:2021 standard [38], the compressive strength and modulus were tested on a testing machine Zwick/Roell Z100 (Zwick Roell, Ulm, Germany) (maximum load-cell capacity 1 kN, the deformation rate: 10%/min). Six cylindrical samples (diameter and height of ~20 mm) for each PUR formulation were cut using a crown drill bit. As compressive strength and modulus are strongly impacted by the foams’ density, these values were adjusted to a single apparent density (40 kg/m^3^). This standardization was performed using the equations outlined by Hawkins et al. [39], enabling a more accurate comparison of results across samples with varying apparent densities.

Rigid PUR foam samples underwent analysis via FTIR spectrometry using a Thermo Scientific Nicolet iS50 spectrometer (Norristown, PA, USA). The analysis was conducted at a resolution of 4 cm^−1^ over 32 scans. FTIR data were acquired through attenuated total reflectance techniques (with ZnSe and diamond crystals).

Water absorption was performed by immersing PUR specimens (dimensions 50 × 50 × 50 mm) into the water for four days, according to ISO 2896:2001 [40].

DSC analysis was conducted using a TA Instrument Mettler Toledo DSC823e under a nitrogen atmosphere (New Castle, DE, USA), with around 6 mg of each sample. The samples were first heated from room temperature to 225 °C at a rate of 10 °C/min, cooled to 25 °C, and then reheated at the same rate to 225 °C. The second heating cycle aimed to ascertain the glass transition temperature.

A Mettler Toledo DMA/SDTA861e instrument was utilized to perform DMA analysis (Columbus, OH, USA). The parameters used were as follows: a temperature range of 25 °C to 220 °C, a ramp rate of 3 °C/min, a frequency of 1 Hz, an amplitude of 5 μm, and a maximum force of 1 N. The mode of compression oscillation was employed. For the experiments, rigid PUR foam samples with dimensions of roughly 13 mm in diameter and 7 mm in height were used. The adjacent-averaging method was used for smoothing in order to address noise in the curves beyond 170 °C.

An autosampler and thermogravimetric analyzer from TA Instruments, the Discovery TGA was used to analyze the samples. The PUR foam samples were put on platinum scale pans and heated at a rate of 10 °C per minute in a nitrogen atmosphere, covering a temperature range of 30 °C to 700 °C. Three or more parallel samples were examined and tested. Software from TA Instruments TRIOS #5.0.0.44608 and OriginPro 2021 9.8.0.200 were used to process the data.

Furthermore, the SA and total renewable material content were computed by dividing the rigid PUR foams’ total mass by the SA mass, or the total mass of the renewable feedstock (SA, water, and tall oil fatty acids). The outcome was given as a weight percentage (wt.%).

## 3. Results and Discussion

### 3.1. Polyol Characterization

Two SA fractions were utilized for polyol synthesis: the SA fraction with increased carboxylic group content (SA-C) was used in synthesis with TOFA, while the SA fraction with increased epoxy group content (SA-E) was employed in synthesis with ETOFA. Synthesis with TOFA was conducted with SA fraction weight ratios of 25 wt.%, 50 wt.%, and 75 wt.%, whereas synthesis with ETOFA followed the same weight ratios.

In the synthesis of SA-C and TOFA-based and SA-E and ETOFA polyols, the primary focus was on achieving polyols with low viscosity, aiming to not exceed 50,000 mPa·s. The desired OH value was in the range between 200 and 500 mg KOH/g, a common OH value often seen for polyols utilized for rigid PUR foam development. A higher OH value is undesirable as it leads to greater consumption of isocyanate, whereas a lower OH value is also undesirable as it fails to yield a highly crosslinked polymer matrix. The acid value of polyols should not surpass 5 mg KOH/g, as exceeding this threshold could lead to technological challenges during rigid PUR foam development. Overall, when identifying the most suitable polyols for the development of rigid PUR foam, the selection criteria prioritized low acid values, OH values within the range of 200–500 mg KOH/g, apparent viscosity below 50,000 mPa·s, and maximizing SA content to the greatest feasible extent.

#### 3.1.1. Polyols from SA Fraction with Increased Carboxylic Group Content

At first, six polyols using 50 wt.% TOFA modifications with respect to SA were synthesized. Polyfunctional alcohols BD, EG, DEG, DEOA, TEOA, or TMP were employed as esterification reagents. Table 6 summarizes the typical characteristics of these six synthesized SA-based polyols, including the acid value, OH value, apparent viscosity, and renewable content.

The acid values of the synthesized polyols were in a range from <5 mg KOH/g to 24 mg KOH/g. Synthesis with bifunctional alcohols yielded products with acid values ranging from 15 mg KOH/g to 24 mg KOH/g. In contrast, polyols synthesized with trifunctional alcohol exhibited significantly lower acid values, dropping below 5 mg KOH/g. Analogous patterns have been reported for SA-based polyol synthesis, where the use of bifunctional alcohols with lower molecular weights tends to yield higher acid values in the final products, contrasting with the synthesis involving trifunctional alcohols [27]. Elevated acid values in polyols can necessitate higher amounts of catalysts during the preparation of PUR systems. This can impact the stability of the polyol system and the quality of resulting rigid PUR foams by influencing their curing behavior and mechanical properties. It is evident that the synthesis with bifunctional alcohols was not particularly suitable for obtaining SA-based polyols. Consequently, further synthesis of SA-based polyols was exclusively conducted using trifunctional alcohols, namely TEOA and TMP.

In subsequent syntheses, TOFA was used at 25 wt.% and 75 wt.% in relation to the mass of SA-C to assess the impact of the ratio on polyol characteristics. Characterizations of these SA-C_TOFA polyols are presented in Table 7.

The ratio of SA-C and TOFA did not affect the acid value, which remained below 5 mg KOH/g, suitable for rigid PUR foams. However, the apparent viscosity increased with higher SA-C content, reaching an unsuitably high viscosity at 75 wt.%. The OH value for synthesized polyols was around 200 mg KOH/g which could be used for rigid PUR foam development; however, taking into account other mentioned pivotal characteristics we decided that these polyols would not be the best choice for further use of these polyols to obtain rigid PUR foam.

Overall, the most promising polyols for rigid PUR production were synthesized using trifunctional alcohols (TEOA or TMP) and did not exceed 50 wt.% of SA-C in the synthesis (SA-C_TOFA(50:50)/TEOA and SA-C_TOFA(50:50)/TMP). This ensured a sufficiently low acid value and apparent viscosity, allowing for excellent mixing capabilities without the need for prior warming when preparing PUR formulations, while maximizing SA content. All of the SA-C_TOFA polyols exhibited moisture content levels lower than 0.2%, making them suited for rigid PUR foam production.

#### 3.1.2. Polyols from SA Fraction with Increased Epoxy Group Content

The trifunctional alcohols (TEOA and TMP) were also selected for synthesizing polyols using SA-E fraction and ETOFA. This choice aimed to ensure a more crosslinked structure with a suitable acid value in the resulting polyols. The ETOFA was used as 25 wt.%, 50 wt.% and 75 wt.% in respect to SA-E mass. The acid value, OH value, apparent viscosities as well as SA content and renewable content of the obtained polyols are presented in Table 8. The acid value of all obtained polyols is below 5 mg KOH/g and the OH value is in a range from 430 mg KOH/g to 500 mg KOH/g.

Polyols modified with ETOFA exhibit higher OH values and higher viscosity compared to those modified with TOFA, indicative of increased crosslinking. Additionally, the higher viscosity observed when using TMP as the alcohol may be influenced by the physical state of the reagent: while TEOA is in a liquid state, TMP is solid at room temperature. The apparent viscosity range using ETOFA modification was from 6570 mPa·s to 1.63 × 10^5^ mPa·s. All SA-E polyols showed a moisture content lower than 0.2%.

SA-E_ETOFA(50:50)/TEOA and SA-E_ETOFA(50:50)/TMP were chosen as the most appropriate higher functional polyols for rigid PUR foam development. This selection was based on their low acid values, similar OH values, and comparable apparent viscosity, all while still offering high SA content.

### 3.2. Multi-Variable Response Surface Model of Rigid PUR Foams

After conducting preliminary experiments with the chosen four most perspective polyols (SA-C_TOFA(50:50)/TEOA and SA-C_TOFA(50:50)/TMP as lower functional polyols; SA-E_ETOFA(50:50)/TEOA and SA-E_ETOFA(50:50)/TMP as higher functional polyols), two polyols were selected for further investigation in the development of MRSM: SA-C_TOFA(50:50)/TMP and SA-E_ETOFA(50:50)/TMP. This choice was made based on the preliminary results, where higher dimensional stability was observed in rigid PUR foams obtained from polyols synthesized with TMP.

Two free-rise rigid PUR foam series (LP and SP) were prepared according to the formulations presented in Section 2.5, Table 3. For each PUR foam series, four variables were optimized: polyol ratios, amounts of physical blowing agent Opteon™ 1100, chemical blowing agent water, and catalyst PC CAT NP10^®^. Five responses were evaluated: apparent density, closed cell content, foaming start time, foam rise time, and shrinkage. The target was to obtain rigid PUR foams with characteristics suitable for thermal insulation. The Analysis of variance (ANOVA) and the equations in terms of actual factors for the LP series and SP series rigid PUR foams are summarized in Tables S2–S6.

**Figure 1:**
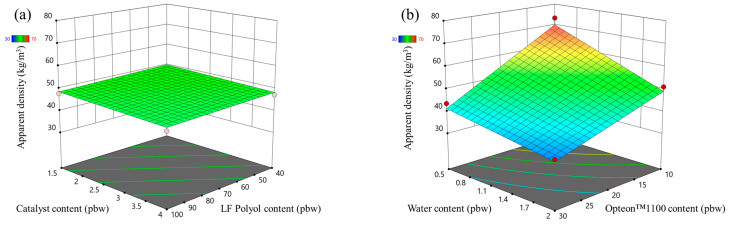
Apparent density response surface for LP series rigid PUR foams: (**a**) Catalyst and LF polyol content influence at Opteon™1100 content = 20 pbw; Water content = 1.25 pbw; (**b**) Water and Opteon™1100 content influence at LF polyol content = 70 pbw; Catalyst content = 2.75 pbw.

**Figure 2:**
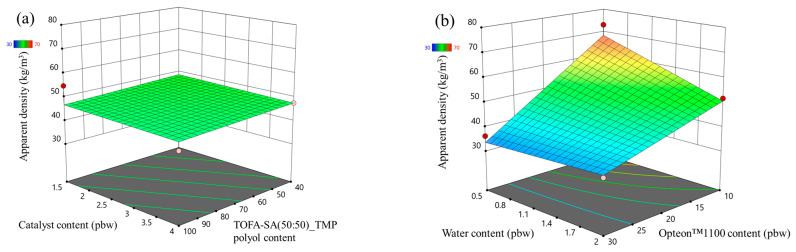
Apparent density response surface for SP series rigid PUR foams: (**a**) Catalyst and TOFA-SA(50:50)_TMP polyol content influence at Opteon™1100 content = 20 pbw; Water content = 1.25 pbw; (**b**) Water and Opteon™1100 content influence at SA-C_TOFA (50:50)/TMP polyol content = 70 pbw; Catalyst content = 2.75 pbw.

The apparent density response surface of LP and SP series PUR foams are depicted in Figure 1 and Figure 2, respectively. In the MRSM graphs, measured data points depicted atop the modeled surface are represented by red dots, while points beneath the surface are indicated by pink dots. Both figures show that the highest influence on the apparent density of the rigid PUR foams was from the two different blowing agents (water and Opteon™1100). The apparent density decreased with the increase in the content of both blowing agents. The catalyst content and LF polyol content had a negligible influence on the apparent density of the developed rigid PUR foams.

**Figure 3:**
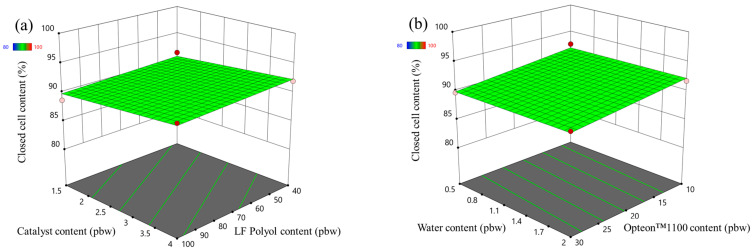
Closed cell content response surface for LP series rigid PUR foams: (**a**) Catalyst and LF polyol content influence at Opteon™1100 content = 20 pbw; Water content = 1.25 pbw; (**b**) Water and Opteon™1100 content influence at LF polyol content = 70 pbw; Catalyst content = 2.75 pbw.

**Figure 4:**
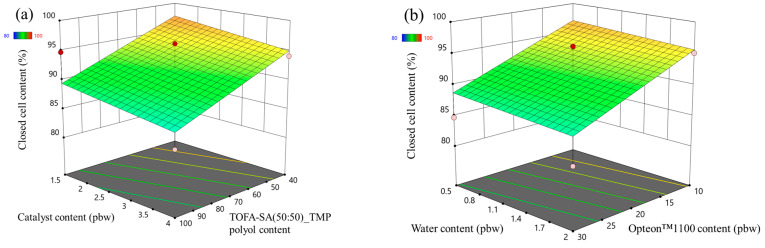
Closed cell content response surface for SP series rigid PUR foams: (**a**) Catalyst and LF polyol content influence at Opteon™1100 content = 20 pbw; Water content = 1.25 pbw; (**b**) Water and Opteon™1100 content influence at LF polyol content = 70 pbw; Catalyst content = 2.75 pbw.

The closed cell content response surface of the LP and SP series rigid PUR foams are depicted in Figure 3 and Figure 4, respectively. In the case of LP series rigid PUR foams the obtained material can be classified as a closed cell foam as the closed cell content was above 90% in the margins of the set boundaries of the experiment. The selected factors had almost no influence on the closed cell content of the LP series rigid PUR foams. However, this was not the case for the SP series rigid PUR foams. The catalyst content exhibited minimal impact on the closed-cell content of the SP series foams, whereas variations in SA-based polyol content notably influenced this parameter, as depicted in Figure 4a. The closed cell content increased with the decrease in the LF SA-based polyol. The relatively low functionality and the steric hindrances of the LF SA-based polyol could contribute to cell opening during the foaming process. The water had an insignificant influence on the closed cell content of the SP series rigid PUR foams but the closed cell content decreased with the increase in the Opteon™1100 content as depicted in Figure 4b. The relatively low apparent density of ~30 kg/m^3^ of the SP series rigid PUR foams at the Opteon™1100 content 30 pbw was the main contributor to the cell rapture during the foaming process. Attaining a high closed-cell content is crucial for producing high-quality rigid PUR foam thermal insulation material. Closed cells facilitate the retention of blowing agent gases within the material’s structure, thereby enhancing its insulating properties. The CO_2_ as well as Opteon™1100 gas has significantly lower thermal conductivity than air which is the main reason for the superior thermal insulation properties of the rigid PUR foams compared to open cell insulation materials like mineral or glass fiber wool.

**Figure 5:**
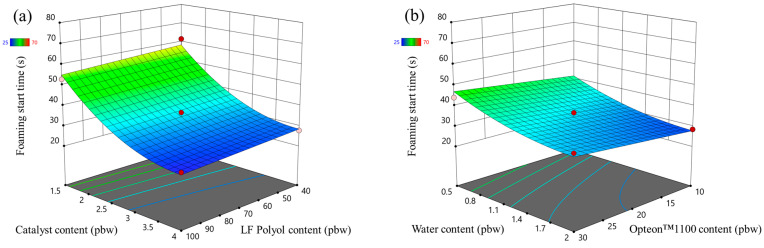
Foaming start time response surface for LP series rigid PUR foams: (**a**) Catalyst and LF polyol content influence at Opteon™1100 content = 20 pbw; Water content = 1.25 pbw; (**b**) Water and Opteon™1100 content influence at LF polyol content = 70 pbw; Catalyst content = 2.75 pbw.

The catalyst content had the largest influence on the foaming start time of the LP series rigid PUR foams as seen in Figure 5a. It decreased with the increase in the catalyst content. The LF polyol and Opteon™1100 content had an insignificant influence on the foaming start time. Furthermore, the foaming start time decreased with the increase in the water content in the LP series rigid PUR foams. The water generates a large amount of heat in the reaction with isocyanate and it promotes faster foaming of the material.

**Figure 6:**
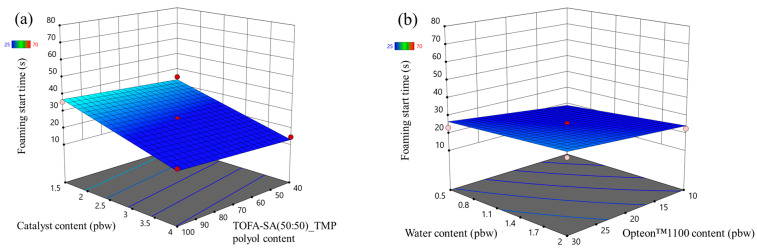
Foaming start time response surface for SP series rigid PUR foams: (**a**) Catalyst and LF polyol content influence at Opteon™1100 content = 20 pbw; Water content = 1.25 pbw; (**b**) Water and Opteon™1100 content influence at LF polyol content content = 70 pbw; Catalyst content = 2.75 pbw.

The foaming parameters were much different for the SP series foams than for the LP series foams. The rigid PUR foam formulations were similar; thus, it is valid to compare both polyol types, i.e., Lupranol and SA-based polyols. Overall, the SP series rigid PUR foams had lower foaming start times than LP series rigid PUR foams as seen in Figure 5 and Figure 6. The faster reactivity of the SP series rigid PUR foams can be explained by the abundance of the primary OH groups whereas Lupranol polyols have only secondary OH groups. The reactivity of the primary OH groups is about three times higher than the secondary OH group reactivity with isocyanate groups [41]. Moreover, the foaming start time of the SP series rigid PUR foams was not affected to the same degree by the amount of the catalyst as in the case of the LP series rigid PUR foams. Refer to Figure 6 and Figure 5, respectively, for visual comparison. This finding holds significant benefit, indicating that SA-based rigid PUR foams can achieve the desired foaming start time with a decreased requirement for catalyst. A smaller amount of catalyst is not only environmentally advantageous but also economically favorable.

**Figure 7:**
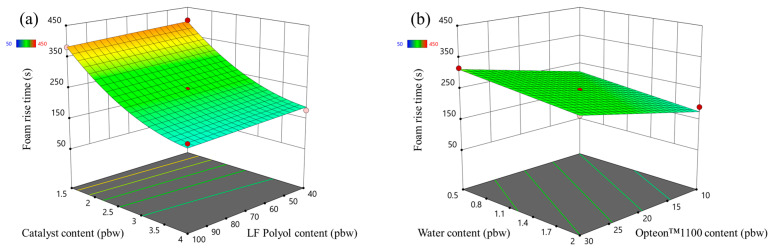
Foam rise time response surface for LP series rigid PUR foams: (**a**) Catalyst and LF polyol content influence at Opteon™1100 content = 20 pbw; Water content = 1.25 pbw; (**b**) Water and Opteon™1100 content influence at LF polyol content = 70 pbw; Catalyst content = 2.75 pbw.

**Figure 8:**
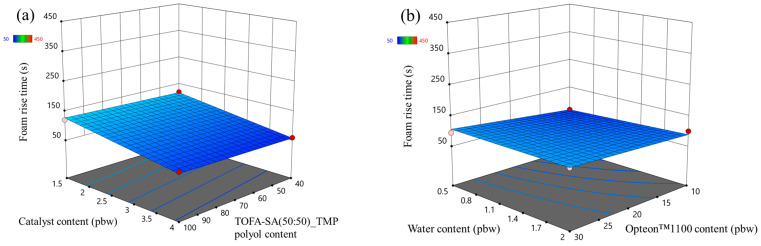
Foam rise time response surface for SP series rigid PUR foams: (**a**) Catalyst and TOFA-SA(50:50)_TMP polyol content influence at Opteon™1100 content = 20 pbw; Water content = 1.25 pbw; (**b**) Water and Opteon™1100 content influence at LF polyol content = 70 pbw; Catalyst content = 2.75 pbw.

The foam rise time response surfaces of the LP series rigid PUR foams and SP series rigid PUR foams are depicted in Figure 7 and Figure 8, respectively. The influence of the changed factors on the foaming rise time was similar to the case of the foaming start time. Foaming rise time decreased with the increase in the catalyst content form as seen in Figure 7a and Figure 8a. The SP series rigid PUR foam rise time was much faster than for LP series rigid PUR foams due to reasons described in the previous paragraph, i.e., primary OH groups.

**Figure 9:**
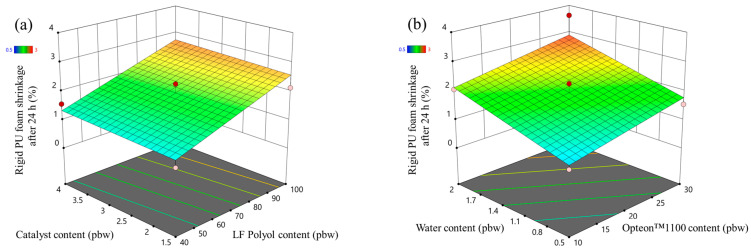
Foam shrinkage after 24 h response surface for LP series rigid PUR foams: (**a**) Catalyst and LF polyol content influence at Opteon™1100 content = 20 pbw; Water content = 1.25 pbw and (**b**) Water and Opteon™1100 content influence at LF polyol content = 70 pbw; Catalyst content = 2.75 pbw.

In the case of LP series rigid PUR foams, the foam shrinkage after 24 h increased with the increase in the content of LF polyol and with the increase in both blowing agents content (Figure 9). The LF polyol Lupranol 3300 decreased the crosslinking density of the PUR polymer matrix which decreases the foam stability. Moreover, the low apparent density of rigid PUR foam also decreases its stability, hence the increase in shrinkage with the increase in the blowing agents. The initial shrinkage of the rigid PUR foams is expected, because the material is hot due to the exothermic foaming reaction and it contracts after cooling down. However, a shrinkage of more than 2% is not desired from a technological point of view as it may cause delamination from the substrate surface when the insulation layer is produced.

**Figure 10:**
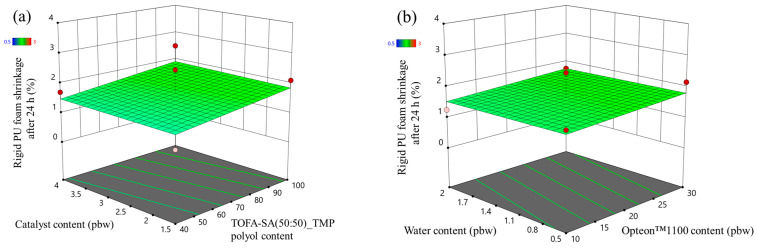
Foam shrinkage after 24 h response surface for SP series rigid PUR foams: (**a**) Catalyst and TOFA-SA(50:50)_TMP polyol content influence at Opteon™1100 content = 20 pbw; Water content = 1.25 pbw and (**b**) Water and Opteon™1100 content influence at LF polyol content = 70 pbw; Catalyst content = 2.75 pbw.

The developed response surface model of the SP series rigid PUR foams shrinkage after 24 h was not valid and the signals could not be distinguished from noise (see Table S6). However, the obtained data still depict the good quality of the SP series rigid PUR foams. The shrinkage after 24 h of most of the foamed samples was below 2% and the changed factors of the designed experiment did not influence the shrinkage (Figure 10). The relatively high OH group functionality and the presence of aromatic groups in the SA-based polyols allowed us to obtain good quality rigid PUR foam material that is well suited for thermal insulation.

### 3.3. Optimization of Rigid PUR Foams’ Formulations

**Figure 11:**
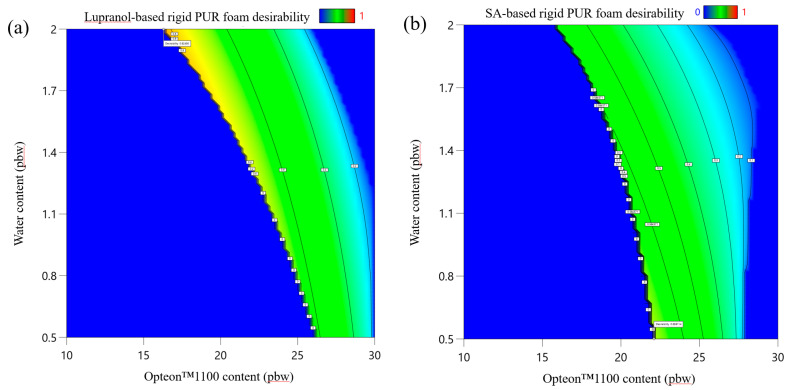
Desirability function of developed rigid PUR foams: (**a**) Lupranol-based polyol formulation at Lupranol 3300 content = 40 pbw and catalyst content = 4.0 pbw; (**b**) SA-based polyol formulation at SA-C_TOFA(50:50)/TMP content = 40 pbw and catalyst content = 1.5 pbw.

The desirability function of LP series rigid PUR foam is depicted in Figure 11a, but the desirability function of SP series polyol rigid PUR foams is depicted in Figure 11b. The main constraint of the rigid PUR foam formulations was the apparent density, which was set from 35 to 45 kg/m^3^ which is the main reason why the desirability function is an area between water and Opteon™ 1100 blowing agent coordinates. The target value of the apparent density of the rigid PUR foams was 45 kg/m^3^. This was selected, as the apparent density in the larger block usually is smaller than in the cup tests; from 40 to 43 kg/m^3^ which is typical for rigid PUR foam thermal insulation material. The three rigid PUR foam formulations with the most desirable characteristics are depicted in Table 9. Larger samples were produced to compare developed thermal insulation and mechanical properties. We chose to select formulations with varied low OH group functionality polyol content (40 pbw and 66 pbw); furthermore, the different content of both blowing agents was selected to compare the properties of the developed material. Lastly, the SA polyols-based rigid PUR foam had significantly less catalyst as these polyols delivered faster foaming parameters. This is beneficial, as catalysts tend to be expensive and there is a risk that catalysts may leak out of the foamed material as VOC emissions. Catalyst leakage is a potential health hazard and should be considered when developing rigid PUR foams.

The optimization of the parameters yielded the desirability function that allows the selection of an optimal rigid PUR foam formulation that will yield material with the most desirable characteristics. As the desirability function is a surface there can be several solutions with equally high desirability value in the margins of error. Thus, we have selected three different rigid PUR foam formulations for each set of experiments with different polyols from the area where desirability functions reach the maximum value.

### 3.4. Characterization of Optimized Rigid PUR Foams

For three formulations of both LP and SP series (Table 9) rigid PUR foams were chosen as the best to reach certain target values (Table 4) according to the created MRS model. Optimized foams were named LPO and SPO foams. The typical characteristics of thermal insulation rigid PUR foams were examined and compared with target values to assess the accuracy of the developed model. Additionally, the results were compared to evaluate SPO foams performance against LPO foams.

**Figure 12:**
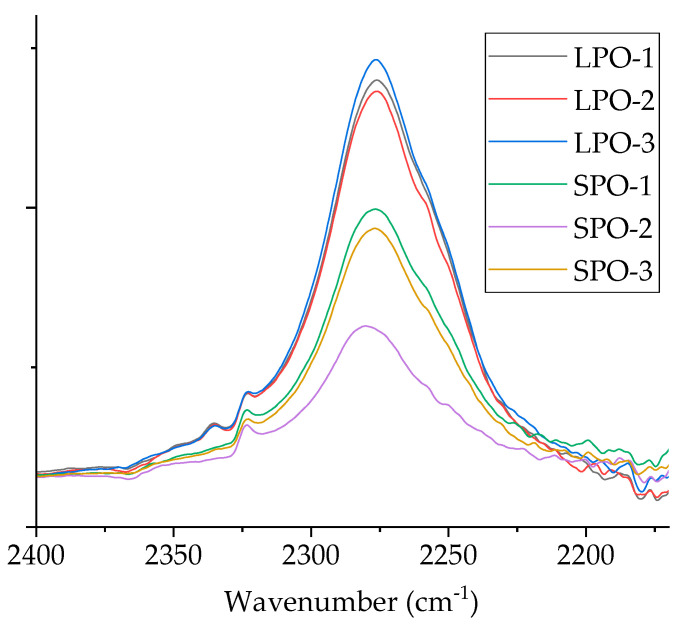
Comparison of isocyanate peak at 2275 cm^−1^ for SPO and LPO foams.

The optimized rigid PUR foams were visually assessed as good-quality products. FTIR analysis (Figure S6) confirmed the formation of the PUR structure. Moreover, SPO foams showed a slightly lower isocyanate peak at 2275 cm^−1^ than LPO foams, indicating a better reaction between SA-based polyols and isocyanate (Figure 12).

A summary of the selected properties of the optimized rigid PUR foams is provided in Table 10. Additionally, the results of compressive strength, modulus, glass transition temperature, and thermal stability will be discussed further.

The apparent density varied in the range of 40.7 kg/m^3^ to 43.5 kg/m^3^, which is close to the chosen target value (45 kg/m^3^). Foaming parameters are satisfactory for free-rise foams. All optimized rigid PUR foams had closed cell contents exceeding 90% indicating a potential application as a thermal insulation material. The SA content in the PUR foams ranged from 13.6% to 14.7%, with the highest concentration observed in the SPO-2 sample. The total renewable material content was remarkably high, ranging from 26.9% to 29.2%. A key characteristic of thermal insulation material is its low thermal conductivity. The values observed were as low as 0.0198–0.0203 W/(m·K) for LPO foams, and even lower for SPO foams, 0.0191–0.0199 W/(m·K). It is considered acceptable for industrial application. Water absorption should also be taken into account. Rigid PUR foams designed for insulation typically aim for a low water absorption rate, often less than 5%, 2%, or even lower. It depends on the specific application and standards. Optimized rigid PUR foams from SA-based polyols showed satisfactory water absorption (~2%), slightly higher than rigid PUR foams from Lupranol polyols.

**Figure 13:**
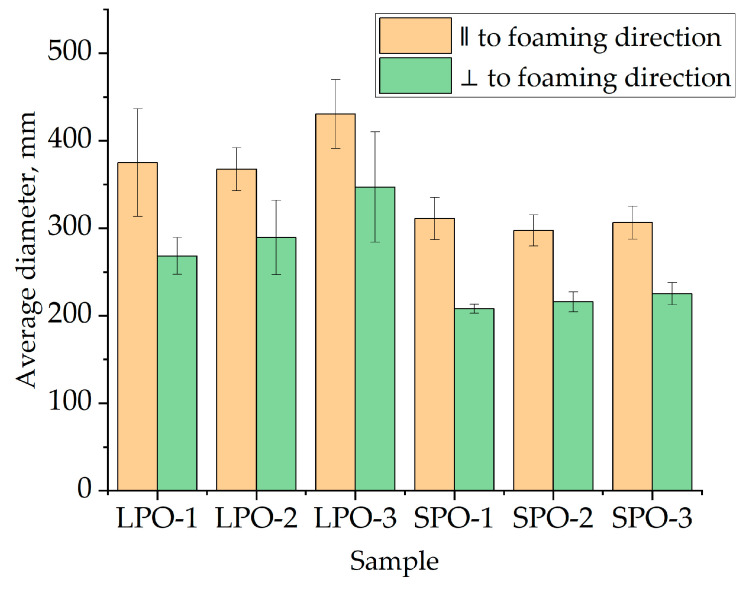
Average cell diameter of optimized rigid PUR foams.

The average cell diameter, as depicted in Figure 13 was determined from optical microscopy images provided in the supplementary data. It is evident that SPO foams exhibited slightly smaller cells, approximately 300 μm parallel to the foaming direction and 200 μm perpendicular to it, compared to LPO foams, which had average cell sizes of approximately 400 μm parallel and 250 μm perpendicular to the foaming direction. The better, i.e., lower thermal conductivity of SPO foams than LPO foams is due to the smaller cell size of the SPO foams.

**Figure 14:**
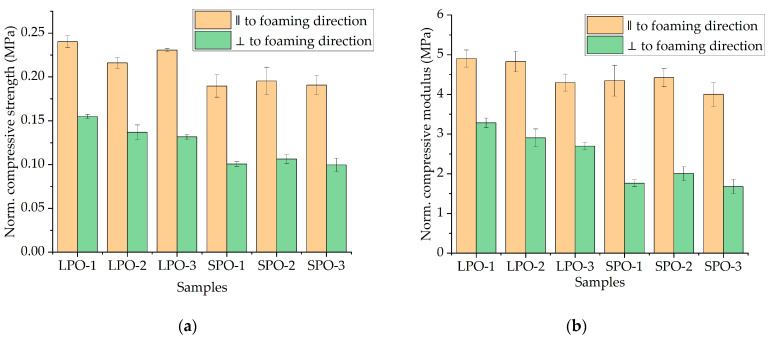
Physical–mechanical properties of the optimized rigid PUR foams: (**a**) normalized compressive strength; (**b**) normalized compressive modulus.

When comparing the compressive properties of rigid PUR foams (as shown in Figure 14) derived from optimized formulations, it is evident that SA-based foams exhibited slightly lower performance than those based on commercially utilized polyols. However, the difference is negligible and results confirm the suitability for thermal insulation applications for all optimized formulations. There were no significant differences in compressive 584 modulus observed between LPO and SPO foams when evaluated parallel to the foaming direction. However, when comparing perpendicular to the foaming direction, SPO foams demonstrated a lower compressive modulus compared to LPO foams.

**Figure 15:**
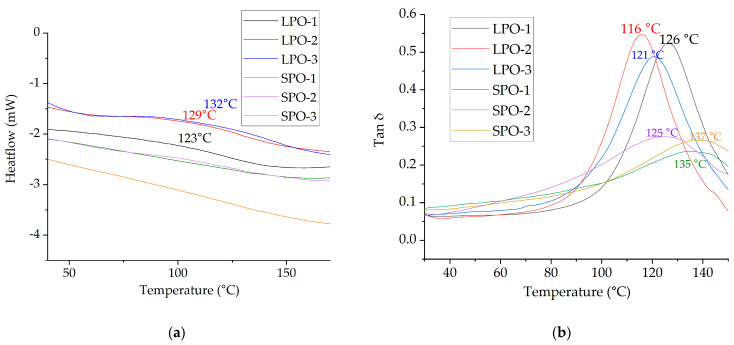
Determinated glass transition temperatures: (**a**) By DSC; (**b**) by DMA from damping factor tan δ results.

DSC and DMA curves are presented in Figure 15. The glass transition temperature for LPO foams was detected by DSC and ranged from 123 °C to 132 °C, while the tan δ peak in DMA occurred from 116 °C to 126 °C. In contrast, the peaks in the damping factor curves for SPO foams were lower and occurred over a wider temperature range compared to LPO foams. It was not possible to detect the glass transition temperature for LPO foams by DSC. Previous studies have reported that SA-based polyols exhibit significant heterogeneity, resulting in an inhomogeneous polymer matrix for rigid PUR foams, leading to a wide range of possible glass transition temperatures and hardly detectable steps by the corresponding DSC equipment [27]. Given that SPO foams contain ~28 wt.% of SA, it seems that the same heterogeneity effect is observed here. Furthermore, modifications with tall oil have shifted the peaks to lower temperatures further from degradation temperature than for rigid PUR foams from SA-based polyols without tall oil [27].

**Figure 16:**
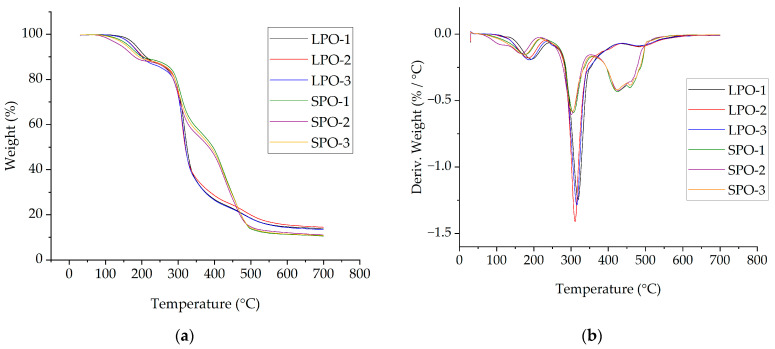
Thermogravimetric analysis of optimized rigid PUR foams: (**a**) TGA curves; (**b**) DTG curves.

Thermal stability was investigated by TGA results. The TGA and DTG curves of the optimized rigid PUR foams, as illustrated in Figure 16, exhibit the typical degradation pattern observed in rigid PUR foams. The thermal properties and associated values are summarized in Table 11. In the case of LPO foams, three distinct degradation steps are evident. During the initial stage, occurring between temperatures of 150 °C to 280 °C, low molecular weight compounds, e.g., TCPP with low decomposition and volatilization temperature, undergo evaporation [42,43]. The most significant degradation occurs during the second step, in the temperature range of 280 °C to 400 °C, with a maximum of around 320 °C. It refers to the release of compounds from soft segment degradation of the urethane network [44,45].

Similarly, SPO foams also demonstrate three main degradation stages, although the second DTG peak at around 305 °C is less pronounced than for LPO foams. Notably, a higher degradation rate is observed during the third stage, manifested as two overlapping peaks above 350 °C, with maximums occurring at approximately 425 °C and 460 °C. It is noteworthy that SPO foams exhibit a lower solid residue weight remaining at 700 °C compared to LPO foams. The observed difference in the third degradation step and the solid residue content at 700 °C likely reflects variances in bond energies stemming from distinct polyol structures [46,47]. Lupranol polyols are polyether polyols, and the C-O-C bond is less stable than the C-C bond in SA-based polyester polyols. Moreover, the SA-based polyols contain polyphenolic aromatic structures, which are released at higher temperatures. The lower solid residue at 700 °C for SPO foams could be attributed to the lower amount of pMDI used in forming foams as the average OH value of SP-series polyols is lower than for LP-series polyols.

## 4. Conclusions

The main goal of this study was to develop and estimate rigid PUR foams from SA polyols modified with TOFA or ETOFA. Sixteen bio-based polyols were synthesized from SA, TOFA, or ETOFA and different alcohols. TEOA and TMP were identified as superior transesterification reagents compared to bifunctional alcohols (BD, EG, and DEG). This distinction was evident as polyols synthesized with trifunctional alcohol demonstrated notably lower acid values, decreasing below 5 mg KOH/g. The optimal weight ratio of SA:TOFA and SA:ETOFA in the synthesized polyols was determined to be 50:50. This ratio enabled the attainment of the highest SA content while maintaining the optimal viscosity of the polyols. In these polyols, the total renewable material content ranged from 70% to 74%, with SA content ranging from 34% to 37%.

Polyols with a SA:TOFA or SA:ETOFA ratio of 50:50, and synthesized with TMP as a transesterification reagent were selected for further investigation in developing the MRSM. Additionally, MRSM was developed for rigid PUR foams obtained from conventional polyols. Created models were used to optimize rigid PUR foam formulations to reach the desired properties of rigid PUR foams. The resulting rigid PUR foams were tested focusing on thermal insulation’s main characteristics to evaluate its potential for use for such an application.

The results, including apparent density (~40–44 kg/m^3^), closed cell content (~95%), compression strength (>0.2 MPa, parallel to the foaming direction), and thermal conductivity (~0.019 W/(m·K)), demonstrated that the bio-based rigid PUR foams from SA and TOFA exhibited competitive qualities in comparison to petroleum-based polyols. The bio-based rigid PUR foam comprised 29.2% renewable materials, including 14.7% SA. These findings highlight the suitability of SA-TOFA polyols for developing rigid PUR foams for sustainable insulation solutions.

## Figures and Tables

**Table 1:** The characteristics of reactants used for SA-based polyol synthesis.

No.	Reactants	Supplier	Characteristics
1	SA fraction with increased carboxylic group content (SA-C)	Synthesized at LSIWC (Riga, Latvia)	Acid value: 100 mg KOH/g Saponification value: 11 mg KOH/g Hydroxyl value (OH value): 120 ± 4 mg KOH/g Apparent viscosity at 25 °C and ẏ = 50 s^−1^: 6.4 × 10^6^ mPa·s
2	SA fraction with increased epoxy group content (SA-E)	Synthesized at LSIWC (Riga, Latvia)	Acid value: 60 mg KOH/g Epoxy group content: 0.068 ± 0.002 mol/100 g OH value: 120 ± 4 mg KOH/g Apparent viscosity at 25 °C and ẏ = 50 s^−1^: not detectable
3	1,4-butanediol (BD)	Sigma Aldrich (St. Louis, MO, USA)	Concentration: >99.0%, reagent plus
4	Ethylene glycol (EG)		99.0%, puriss
5	Diethylene glycol (DEG)		99.0%, puriss
6	Diethanolamine (DEOA)		≥98.5%, ACS reagent
7	Triethanolamine (TEOA)		98.0%, reagent grade
8	Trimethylolpropane (TMP)		>99.0%, reagent plus
9	Potassium hydroxide		85%, reagent grade
10	Tall oil fatty acids (TOFA)	Forchem Oyj (Rauma, Finland)	Fatty acid content: ≥96% Iodine value: 193 ± 2 g I_2_/100 g Acid value: 155 ± 2 mg KOH/g
11	Epoxidized tall oil fatty acids (ETOFA)	Synthesized at LSIWC (Riga, Latvia)	Iodine value: 27 ± 2 g I_2_/100 g Acid value: 140 ± 2 mg KOH/g Epoxy value: 0.24 ± 0.02

**Table 2:** Evaluated characteristics and methods used in accordance.

Characteristic	Method
Acid value	DIN EN ISO 2114:2002-06 [28]
OH value	DIN EN ISO 4629-2:2016 [29]
Iodine value	ISO 3961:2013 [30]
Oxirane value	ASTM D1652-04:2004 [31]
Apparent viscosity	DIN 53019:2008 [32]

**Table 3:** Rigid PUR foam formulations for MRSM.

Ingredient		Weight, Parts by Weight (pbw)	
		LP Series	SP Series
Polyols ^1^	SA-C_TOFA(50:50)/TMP	-	40–100
	SA-E_ETOFA(50:50)/TMP	-	0–60
	Lupranol 3300	40–100	-
	Lupranol 3422	0–60	-
Blowing agents	Total water	1.25–2	1.25–2
	Opteon™ 1100	10–30	10–30
Catalysts	Polycat NP10	1.5–4	1.5–4
	PC CAT TKA 30	0.5	0.5
Surfactant	L-6915	2.5	2.5
Flame retardant	TCPP ^2^	21.6–25.5	18.7–23.3
Isocyanate	pMDI ^3^	123–165	89–140

^1^ Polyols sum was 100 g. ^2^ Weight of TCPP was varied to be 8 wt.% from the total foam mass. ^3^ Weight of pMDI was varied to reach an NCO/OH ratio of 1.2.

**Table 4:** Optimized parameters for the rigid PUR foams of LP series, the lower and upper limit of the parameter, and the importance level of the parameter optimization.

Name	Goal	Lower Limit	Upper Limit	Lower Weight	Upper Weight	Importance
A: LF polyol Content	is in range	40	100	1	1	3
B: Opteon 1100 content	minimize	10	30	1	1	3
C: Water content	is in range	0.5	2	1	1	3
D: Catalyst content	is in range	1.5	4	1	1	3
Apparent density	is target = 45	35	45	1	1	5
Closed cell content	maximize	89	93	1	1	3
Start time	none	23	40	1	1	3
Rise time	none	152	463	1	1	3
Shrinkage	minimize	0.6	3.6	1	1	3
Renewable materials content	none	7.5	8.6	1	1	5

**Table 5:** Optimized parameters for the rigid PUR foams of SP series, the lower and upper limit of the parameter, and the importance level of the parameter optimization.

Name	Goal	Lower Limit	Upper Limit	Lower Weight	Upper Weight	Importance
A: LF polyol Content	is in range	40	100	1	1	3
B: Opteon 1100 content	minimize	10	30	1	1	3
C: Water content	is in range	0.5	2	1	1	3
D: Catalyst content	is in range	1.5	4	1	1	3
Apparent density	is target = 45	35	45	1	1	5
Closed cell content	maximize	82	97	1	1	3
Start time	none	14	47	1	1	3
Rise time	none	53	166	1	1	3
Shrinkage	minimize	0.95	2.5	1	1	3
Renewable materials content	maximize	25.4	32	1	1	5

**Table 6:** Characteristics of polyols synthesized from SA-C and TOFA at weight ratio 50:50 using different polyfunctional alcohols (BD, EG, DEG, DEOA, TEOA, and TMP).

	SA-C_TOFA (50:50)/BD	SA-C_TOFA (50:50)/EG	SA-C_TOFA (50:50)/DEG	SA-C_TOFA (50:50)/DEOA	SA-C_TOFA (50:50)/TEOA	SA-C_TOFA (50:50)/TMP
Acid value, mg KOH/g	15 ± 2	24 ± 2	21 ± 3	20 ± 2	<5	<5
OH value, mg KOH/g	169 ± 6	191 ± 11	171 ± 4	261 ± 11	248 ± 8	232 ± 6
Apparent viscosity ^1^, mPa∙s	1.87 × 10^2^	2.93 × 10^2^	1.21 × 10^2^	7.89 × 10^2^	1.42 × 10^3^	2.69 × 10^3^
SA content, %	40	43.1	38.9	38.9	35.7	37
Renewable content, %	80	86.2	77.8	77.8	71.4	74

^1^ at 25 °C, (ẏ = 50 s^−1^).

**Table 7:** Characteristics of polyols synthesized from SA-C and TOFA at weight ratios of 25:75 and 75:25 using different polyfunctional alcohols (TEOA and TMP).

	SA-C_TOFA (25:75)/TEOA	SA-C_TOFA (25:75)/TMP	SA-C_TOFA (75:25)/TEOA	SA-C_TOFA (75:25)/TMP
Acid value, mg KOH/g	<5	<5	<5	<5
OH value, mg KOH/g	234 ± 4	254 ± 3	193 ± 2	228 ± 2
Apparent viscosity ^1^, mPa∙s	4.15 × 10^2^	9.90 × 10^2^	6.9 × 10^5^	2.3 × 10^5^
SA content, %	17.1	17.7	56.1	57.5
Renewable content, %	68.5	70.7	74.8	76.7

^1^ at 25 °C, (ẏ = 50 s^−1^).

**Table 8:** Characteristics of polyols synthesized from SA-C and TOFA using different polyfunctional alcohols.

	SA-E_ETOFA (25:75)/TEOA	SA-E_ETOFA (25:75)/TMP	SA-E_ETOFA (50:50)/TEOA	SA-E_ETOFA (50:50)/TMP	SA-E_ETOFA (75:25)/TEOA	SA-E_ETOFA (75:25)/TMP
Acid value, mg KOH/g	<5	<5	<5	<5	<5	<5
OH value, mg KOH/g	490 ± 3	452 ± 14	426 ± 8	430 ± 11	470 ± 15	500 ± 11
Apparent viscosity *, mPa∙s	6.57 × 10^3^	3.99 × 10^4^	1.40 × 10^4^	3.89 × 10^4^	1.06 × 10^5^	1.63 × 10^5^
SA content, %	15.9	16.5	34.1	35.2	54.6	56.1
Renewable content, %	63.7	66	68.2	70.4	72.8	74.8

* at 25 °C, (ẏ = 50 s^−1^).

**Table 9:** Optimized rigid PUR foam formulations.

Ingredient		Weight, g					
		LP Series			SP Series		
		LPO-1	LPO-2	LPO-3	SPO-1	SPO-2	SPO-3
Polyols	SA-C_TOFA(50:50)/TMP	-	-	-	40	67	40
	SA-E_ETOFA(50:50)/TMP	-	-	-	60	33	60
	Lupranol 3300	40	66	40	-	-	-
	Lupranol 3422	60	34	60	-	-	-
Blowing agents	Total water	2.0	2.0	1.1	0.5	0.5	0.74
	Opteon™ 1100	16.3	14.8	23.6	24.3	24.3	23.6
Catalysts	Polycat NP10	4	4	3.8	1.55	1.55	3.03
	PC CAT TKA 30	0.5	0.5	0.5	0.5	0.5	0.5
Surfactant	L-6915	2.5	2.5	2.5	2.5	2.5	2.5
Flame retardant	TCPP ^1^	25.3	24.5	24.5	21	20	21.5
Isocyanate	pMDI ^2^	165	158	149	113	102	117

^1^ Weight of TCPP was adjusted to be 8 wt.% from the total foam mass. ^2^ Weight of pMDI was varied to reach an NCO/OH ratio of 1.2.

**Table 10:** Selected properties of optimized rigid PUR foams.

	LPO-1	LPO-2	LPO-3	SPO-1	SPO-2	SPO-3
Foaming start time, s	25	24	34	43	57	29
Foam gel time, s	103	104	137	67	90	47
Tack-free time, s	196	196	219	88	114	62
Foam rise time, s	186	185	205	120	129	110
Apparent density, kg/m^3^	43.2	43.5	43.4	43.9	40.7	43.2
Closed cell content, vol.%	94	93	96	96	95	95
SA content, %	0	0	0	13.8	14.7	13.6
Renewable materials, %	8.2	8.0	8.2	27.4	29.2	26.9
Thermal conductivity, mW/(m·K) (0…20 °C)	19.84	20.26	20.26	19.97	19.07	19.34
Geometrical anisotropy	1.4	1.3	1.2	1.5	1.4	1.4
Water absorption, V%	1.69 ± 0.09	1.71 ± 0.07	1.92 ± 0.01	2.02 ± 0.25	2.16 ± 0.06	1.94 ± 0.04

**Table 11:** Thermal properties of the optimized rigid PUR foams.

	LPO-1	LPO-2	LPO-3	SPO-1	SPO-2	SPO-3
First Onset ^1^, °C	162 ± 2	148 ± 1	149 ± 1	138 ± 3	149 ± 4	130 ± 1
T_m5%_ ^2^, °C	183 ± 1	174 ± 1	173 ± 1	165 ± 1	139 ± 4	158 ± 1
T_m10%_ ^2^, °C	211 ± 1	205 ± 1	200 ± 1	203 ± 2	178 ± 2	194 ± 1
Residue ^3^, %	13.7 ± 0.1	14.5 ± 0.1	13.7 ± 0.4	10.6 ± 0.2	10.6 ±0.5	10.7 ± 0.2
T_max1_ ^4^, °C	195 ± 1	185 ± 1	189 ± 1	177 ± 1	167 ± 2	174 ± 1
T_max2_ ^4^, °C	319 ± 1	311 ± 1	316 ± 1	306 ± 1	301 ± 1	303 ± 1
T_max3_ ^4^, °C	483 ± 1	488 ± 1	490 ± 5	458 ± 3	458 ± 1	459 ± 1

^1^ Thermal degradation onset temperature. ^2^ Temperature at a weight loss of 5% and 10%. ^3^ Weight of the solid residue remaining at 700 °C. ^4^ Three main DTG peaks.

## Data Availability

Data are contained within the article and Supplementary Materials.

## Supplementary Materials

The following supporting information can be downloaded at: https://www.mdpi.com/article/10.3390/polym16070942/s1, Figure S1: Parity plot of the apparent density parameter response surface models: a—LP series rigid PUR foams; b—SP series rigid PUR foams; Figure S2: Parity plot of the apparent density parameter response surface models: a—LP series rigid PUR foams; b—SP series rigid PUR foams; Figure S3: Parity plot of the foaming start time parameter response surface models: a—LP series foams; b –SP series foams; Figure S4: Parity plot of the foam rise time parameter response surface models: a—LP series; b –SP series. Figure S5: Parity plot of the foam shrinkage after 24 h parameter response surface models: a—LP series; b—SP series; Figure S6: FTIR curves of LPO and SPO foams. Figure S7. Optical microscopy images of SPO and LPO foams, captured in both parallel (||) and perpendicular (⊥) directions to the foaming directions. Table S1: Different runs/formulations for LP series and SP series rigid PUR foams; Table S2: ANOVA and the equation in terms of actual factors for the LP series and SP series rigid PUR foams apparent density parameter response surface models and the R^2^ values of the model; Table S3: ANOVA and the equation in terms of actual factors for the LP series and SP series rigid PUR foams closed cell content parameter response surface models and the R^2^ values of the model; Table S4: ANOVA and the equation in terms of actual factors for the LP series and SP series rigid PUR foams foaming start time parameter response surface models and the R^2^ values of the model; Table S5: ANOVA and the equation in terms of actual factors for the LP series and SP series rigid PUR foams rise time parameter response surface models and the R^2^ values of the model; Table S6: ANOVA and the equation in terms of actual factors for the LP series and SP series rigid PU foams shrinkage after 24 h response surface models and the R^2^ values of the model.

## References

[1] Backes J.G., Traverso M. (2022). Life Cycle Sustainability Assessment as a Metrics towards SDGs Agenda 2030. Curr. Opin. Green Sustain. Chem..

[2] Ma Y., Xiao Y., Zhao Y., Bei Y., Hu L., Zhou Y., Jia P. (2022). Biomass Based Polyols and Biomass Based Polyurethane Materials as a Route towards Sustainability. React. Funct. Polym..

[3] Tuladhar R., Yin S. (2019). Sustainability of Using Recycled Plastic Fiber in Concrete. Use of Recycled Plastics in Eco-Efficient Concrete.

[4] Simón D., Borreguero A.M., de Lucas A., Gutiérrez C., Rodríguez J.F., Jiménez E., Cabañas B., Lefebvre G. (2015). Sustainable Polyurethanes: Chemical Recycling to Get It. Environment, Energy and Climate Change I Environmental Chemistry of Pollutants and Wastes.

[5] Tran H.T.T., Deshan A.D.K., Doherty W., Rackemann D., Moghaddam L. (2022). Production of Rigid Bio-Based Polyurethane Foams from Sugarcane Bagasse. Ind. Crops Prod..

[6] Mazar A., Paleologou M. (2023). New Approach to Recycle and Valorize the First Filtrate of the LignoForce System^TM^: Lignin Extraction and Its Use in Rigid Lignin-Based Polyurethane Foams. Int. J. Biol. Macromol..

[7] Ivdre A., Soto G.D., Cabulis U. (2016). Polyols Based on Poly(Ethylene Terephthalate) and Tall Oil: Perspectives for Synthesis and Production of Rigid Polyurethane Foams. J. Renew. Mater..

[8] Patil C.K., Jirimali H.D., Paradeshi J.S., Chaudhari B.L., Alagi P.K., Mahulikar P.P., Hong S.C., Gite V.V. (2021). Chemical Transformation of Renewable Algae Oil to Polyetheramide Polyols for Polyurethane Coatings. Prog. Org. Coatings.

[9] Phung Hai T.A., Neelakantan N., Tessman M., Sherman S.D., Griffin G., Pomeroy R., Mayfield S.P., Burkart M.D. (2020). Flexible Polyurethanes, Renewable Fuels, and Flavorings from a Microalgae Oil Waste Stream. Green Chem..

[10] Polaczek K., Kurańska M., Auguścik-Królikowska M., Prociak A., Ryszkowska J. (2021). Open-Cell Polyurethane Foams of Very Low Density Modified with Various Palm Oil-Based Bio-Polyols in Accordance with Cleaner Production. J. Clean. Prod..

[11] Hsieh C.C., Chen Y.C. (2020). Synthesis of Bio-Based Polyurethane Foam Modified with Rosin Using an Environmentally-Friendly Process. J. Clean. Prod..

[12] Sardon H., Mecerreyes D., Basterretxea A., Avérous L., Jehanno C. (2021). From Lab to Market: Current Strategies for the Production of Biobased Polyols. ACS Sustain. Chem. Eng..

[13] Hai T.A.P., Tessman M., Neelakantan N., Samoylov A.A., Ito Y., Rajput B.S., Pourahmady N., Burkart M.D. (2021). Renewable Polyurethanes from Sustainable Biological Precursors. Biomacromolecules.

[14] Vevere L., Fridrihsone A., Kirpluks M., Cabulis U. (2020). A Review of Wood Biomass-Based Fatty Acids and Rosin Acids Use in Polymeric Materials. Polymers.

[15] Rizhikovs J., Zandersons J., Dobele G., Paze A. (2015). Isolation of Triterpene-Rich Extracts from Outer Birch Bark by Hot Water and Alkaline Pre-Treatment or the Appropriate Choice of Solvents. Ind. Crops Prod..

[16] Gosecki M., Urbaniak M., Makarewicz C., Gosecka M. (2024). Converting Unrefined Birch Suberin Monomers into Vitrimer. ACS Sustain. Chem. Eng..

[17] Heinämäki J., Pirttimaa M.M., Alakurtti S., Pitkänen H.P., Kanerva H., Hulkko J., Paaver U., Aruväli J., Yliruusi J., Kogermann K. (2017). Suberin Fatty Acids from Outer Birch Bark: Isolation and Physical Material Characterization. J. Nat. Prod..

[18] Sousa A.F., Gandini A., Caetano A., Maria T.M.R., Freire C.S.R., Neto C.P., Silvestre A.J.D. (2016). Unravelling the Distinct Crystallinity and Thermal Properties of Suberin Compounds from Quercus Suber and Betula Pendula Outer Barks. Int. J. Biol. Macromol..

[19] Sousa A.F., Gandini A., Silvestre A.J.D., Neto C.P., Cruz Pinto J.J.C., Eckerman C., Holmbom B. (2011). Novel Suberin-based Biopolyesters: From Synthesis to Properties. J. Polym. Sci. Part A Polym. Chem..

[20] Gandini A., Pascoal Neto C., Silvestre A.J.D. (2006). Suberin: A Promising Renewable Resource for Novel Macromolecular Materials. Prog. Polym. Sci..

[21] Rizikovs J., Godina D., Makars R., Paze A., Abolins A., Fridrihsone A., Meile K., Kirpluks M. (2021). Suberinic Acids as a Potential Feedstock for Polyol Synthesis: Separation and Characterization. Polymers.

[22] Pinto P.C.R.O., Sousa A.F., Silvestre A.J.D., Neto C.P., Gandini A., Eckerman C., Holmbom B. (2009). Quercus Suber and Betula Pendula Outer Barks as Renewable Sources of Oleochemicals: A Comparative Study. Ind. Crops Prod..

[23] Rizhikovs J., Brazdausks P., Paze A., Tupciauskas R., Grinins J., Puke M., Plavniece A., Andzs M., Godina D., Makars R. (2022). Characterization of Suberinic Acids from Birch Outer Bark as Bio-Based Adhesive in Wood Composites. Int. J. Adhes. Adhes..

[24] Paze A., Rizhikovs J. (2019). Study of an Appropriate Suberinic Acids Binder for Manufacturing of Plywood. Key Eng. Mater..

[25] Cordeiro N., Belgacem M.N., Gandini A., Neto C.P. (1997). Urethanes and Polyurethanes from Suberin: 1. Kinetic Study. Ind. Crops Prod..

[26] Cordeiro N., Belgacem M.N., Gandini A., Pascoal Neto C. (1999). Urethanes and Polyurethanes from Suberin 2: Synthesis and Characterization. Ind. Crops Prod..

[27] Ivdre A., Abolins A., Volkovs N., Vevere L., Paze A., Makars R., Godina D., Rizikovs J. (2023). Rigid Polyurethane Foams as Thermal Insulation Material from Novel Suberinic Acid-Based Polyols. Polymers.

[28] (2000). Plastics (Polyester Resins) and Paints and Varnishes (Binders)—Determination of Partial Acid Value and Total Acid Value.

[29] (2016). Binders for Paints and Varnishes—Determination of Hydroxyl Value—Part 2: Titrimetric Method Using a Catalyst.

[30] (2013). Animal and Vegetable Fats and Oils Determination of Iodine Value.

[31] (2004). Standard Test Method for Epoxy Content of Epoxy Resins.

[32] (2008). Viscometry—Measurement Of Viscosities And Flow Curves By Means of Rotational Viscometers—Part 1: Principles And Measuring Geometry.

[33] Myers R.H., Montgomery D.C., Anderson-Cook C.M. (2016). Response Surface Methodology: Process and Product Optimization Using Designed Experiments.

[34] (2006). Cellular Plastics and Rubbers—Determination of Apparent Density.

[35] (2016). Rigid Cellular Plastics—Determination of the Volume Percentage of Open Cells and of Closed Cells.

[36] (1991). Thermal Insulation—Determination of Steady-State Thermal Resistance and Related Properties—Heat Flow Meter Apparatus.

[37] (2020). Standard Test Method for Cell Size of Rigid Cellular Plastics.

[38] (2021). Rigid Cellular Plastics—Determination of Compression Properties.

[39] Hawkins M.C., O’Toole B., Jackovich D. (2005). Cell Morphology and Mechanical Properties of Rigid Polyurethane Foam. J. Cell. Plast..

[40] (2001). Rigid Cellular Plastics—Determination of Water Absorption.

[41] Ionescu M. (2005). Chemistry and Technology for Polyurethane.

[42] van der Veen I., de Boer J. (2012). Phosphorus Flame Retardants: Properties, Production, Environmental Occurrence, Toxicity and Analysis. Chemosphere.

[43] Xu D., Yu K., Qian K. (2018). Thermal Degradation Study of Rigid Polyurethane Foams Containing Tris(1-Chloro-2-Propyl)Phosphate and Modified Aramid Fiber. Polym. Test..

[44] Jabar J.M. (2022). Production of Sustainable Rigid Polyurethane Foam from Chemically Modified Underutilized Jatropha Curcas L Seed Oil: Influence of Polyol Chemical Structure on Properties of Polymer. Curr. Res. Green Sustain. Chem..

[45] Kim B.H., Yoon K., Moon D.C. (2012). Thermal Degradation Behavior of Rigid and Soft Polyurethanes Based on Methylene Diphenyl Diisocyanate Using Evolved Gas Analysis-(Gas Chromatography)-Mass Spectrometry. J. Anal. Appl. Pyrolysis.

[46] Elbers N., Ranaweera C.K., Ionescu M., Wan X., Kahol P.K., Gupta R.K. (2017). Synthesis of Novel Biobased Polyol via Thiol-Ene Chemistry for Rigid Polyurethane Foams. J. Renew. Mater..

[47] Narine S.S., Kong X., Bouzidi L., Sporns P. (2007). Physical Properties of Polyurethanes Produced from Polyols from Seed Oils: I. Elastomers. J. Am. Oil Chem. Soc..

