# Supplementary material for: Rigid Polyurethane Foams’ Development and Optimization from Polyols Based on Depolymerized Suberin and Tall Oil Fatty Acids
Source: Polymers (Basel). 2024 Mar 29;16(7):942. doi: 10.3390/polym16070942 (PMC11013755; doi:10.3390/polym16070942)
Supplement: Supplementary file 1 [file polymers-16-00942-s001.zip › polymers-2918374-supplementary.pdf]

Suplementary information

Table S1 describes the changed factors and their coded levels, where 0 value is attributed to the coded mean value and +1 and -1 are attributed to the coded high and coded low values of the changed factors, respectively. The content of the low functionality (LF) polyol was changed between 40 pbw to 100 pbw and the content of the high functionality polyol was changed between 0 pbw to 60 pbw. The bio-based polyol SA-C_TOFA (50:50)/TMP polyol was selected as LF polyol, synthesised from SA and tall oil fatty acids. It was compared to the commercially available Lupranol 3300 which OH group functionality is ~3. Whereas the high functional bio-based polyol SA-E_ETOFA (50:50)/TMP was selected and compared to the Lupranol 3422. Two different blowing agent content influence on the rigid PUR foam properties were investigated, physical blowing agent Opteon™ 1100 and water as the chemical blowing agent. The Opteon™ 1100 content was changed between 10-30 pbw and the water content was changed between 1.25-2.00 pbw. Lastly, the gelling catalyst Polycat NP10 content influence on the rigid PUR properties was investigated between 1.5-4.0 pbw.

Table S1. Different runs/formulations for LP series and SP series rigid PUR foams

Run	LF polyol content, pbw	Coded level	Opteon™ 1100 content, pbw	Coded level	Water content, pbw	Coded level	Catalyst content, pbw	Coded level
1	100	+1	10	-1	1.25	0	2.75	0
2	70	0	20	0	1.25	0	2.75	0
3	70	0	30	+1	1.25	0	1.50	-1
4	70	0	20	0	2.00	+1	1.50	-1
5	40	-1	20	0	0.50	-1	2.75	0
6	100	+1	20	0	1.25	0	1.50	-1
7	40	-1	30	+1	1.25	0	2.75	0
8	70	0	20	0	2.00	+1	4.00	+1
9	70	0	10	-1	2.00	+1	2.75	0
10	70	0	20	0	1.25	0	2.75	0
11	70	0	10	-1	1.25	0	4.00	+1
12	70	0	10	-1	0.50	-1	2.75	0
13	100	+1	30	+1	1.25	0	2.75	0
14	100	+1	20	0	1.25	0	4.00	+1
15	70	0	30	+1	0.50	-1	2.75	0
16	70	0	20	0	0.50	-1	1.50	-1
17	70	0	30	+1	2.00	+1	2.75	0
18	70	0	20	0	1.25	0	2.75	0
19	70	0	20	0	0.50	-1	4.00	+1
20	100	-1	20	0	2.00	+1	2.75	0
21	70	0	30	+1	1.25	0	4.00	+1
22	40	-1	20	0	2.00	+1	2.75	0
23	40	-1	20	0	1.25	0	4.00	+1
24	100	+1	20	0	0.50	-1	2.75	0
25	40	0	10	-1	1.25	0	2.75	0
26	40	0	20	0	1.25	0	1.50	-1
27	70	-1	10	-1	1.25	0	1.50	-1

Apparent density MRSM

The LF polyol, Opteon™1100, water, and catalyst content influence on the apparent density of developed two sets of rigid PUR foams was approximated by a two factor interaction (2FI) model and the ANOVA of the models is summarised in Table S2. The Model F-value of 62.23 and 32.24 for LP series foams and SP series foams, respectively, implies that the models are significant and there is only a 0.01% chance that an F-values this large could occur due to noise. Adequate precision measures the signal to noise ratio and it was 29.704 and 20.901 for LP foams and SP foams, respectively, which indicates an adequate signal. Developed models can be used to navigate the design space. The parity plots of experimental results and modelled apparent density of the developed rigid PUR foam are depicted in Figure S1 and they show a good fit of the developed model.

Table S2. ANOVA and the equation in terms of actual factors for the LP series and SP series rigid PUR foams apparent density parameter response surface models and the R^2 values of the model.

Factors	Symbol	Apparent density equation LP series	F-value	Sum of Squares	Apparent density equation SP series	F-value	Sum of Squares
Model			62.23	2174.66		32.24	1945.43
Intercept		+109.235			+98.667		
LF polyol content	A	-0.198			-0.0317		
Opteon™1100 content	B	-2.264			-2.083		
Water content	C	-18.316			-18.650		
Catalyst content	D	-0.675			-0.450		
	A·B	+0.0087					
	B·C	+0.437			+0.677		
R^2		0.934			0.862		
Adequate Precision		29.704			20.901		

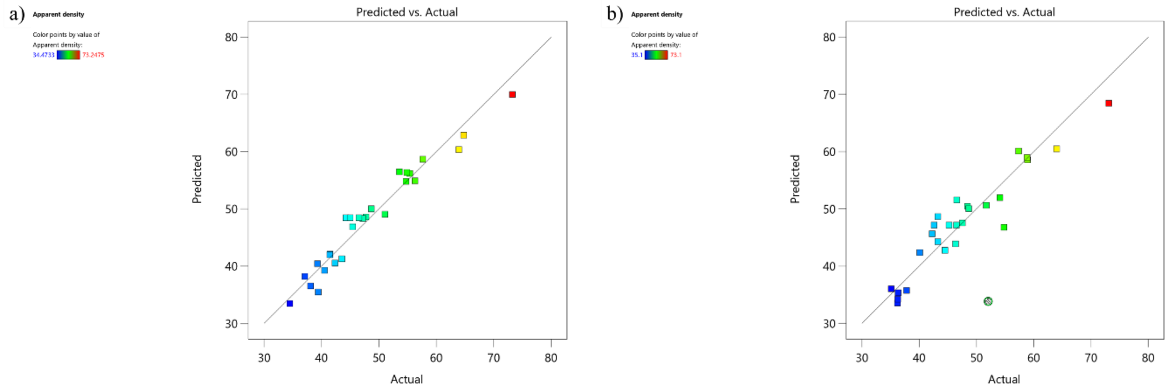

Figure S1. Parity plot of the apparent density parameter response surface models: **a** – LP series rigid PUR foams; **b** – SP series rigid PUR foams.

Closed cell MRSM

The LF polyol content, Opteon™1100 content, water content and catalyst content influence on the closed cell content of developed two sets of rigid PUR foams was approximated by a linear model and the ANOVA of the models is summarised in Table S3. The Model F-value of 8.76 and 4.11 for LP series foams and SP series, respectively, and it implies that the models are significant and there is only a 0.02% and 1.54% chance that an F-values this large could occur due to noise. Adequate precision measures the signal to noise ratio and it was 10.548 and 5.863 for LP foams and SP foams, respectively, which indicates an adequate signal. Developed models can be used to navigate the design space. The parity plots of the experimental results and modelled closed cell content of the developed rigid PUR foam are depicted in Figure S2 and they show a decent fit of the developed model.

Table S3. ANOVA and the equation in terms of actual factors for the LP series and SP series rigid PUR foams closed cell content parameter response surface models and the R^2 values of the model.

**Factors**	**Symbol**	**Closed cell content equation LP series**	**F-value**	**Sum of Squares**	**Closed cell content equation SP series**	**F-value**	**Sum of Squares**
Model			8.76	23.82		4.11	234.66
Intercept		+92.271			+114.161		
LF polyol content	A	-0.0145			-0.137		
Opteon™1100 content	B	-0.107			-0.414		
Water content	C	+0.132			-0.928		
Catalyst content	D	+0.642			-1.076		
R^2		0.544			0.361		
Adequate Precision		10.548			5.863		

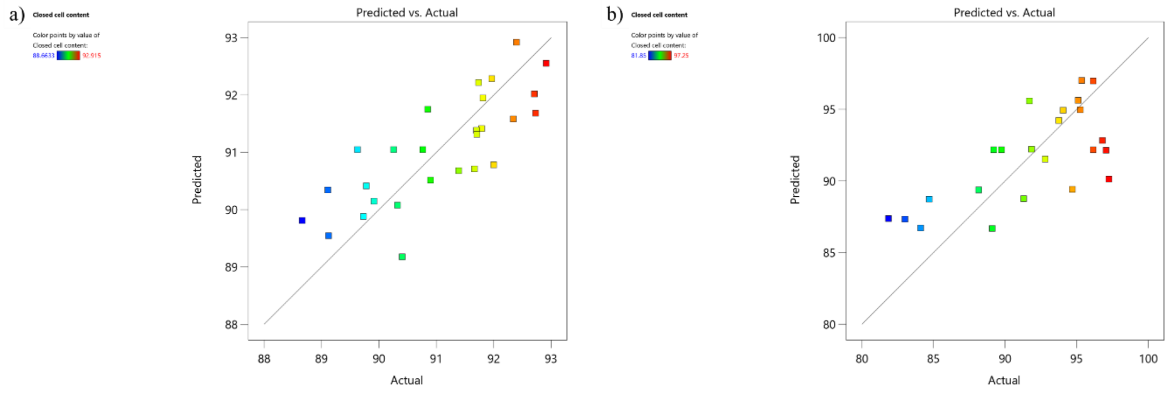

Figure S2. Parity plot of the apparent density parameter response surface models: **a** – LP series rigid PUR foams; **b** – SP series rigid PUR foams.

Foaming start time MRSM

The LF polyol content, Opteon™1100 content, water content and catalyst content influence on the foaming start time of developed two sets of rigid PUR foams was approximated by a quadratic and 2FI models, respectively, and the ANOVA of the models is summarised in Table S4. The Model F-value of 99.23 for LP series foams and 45.23 for SP series foams implies that the models are significant and there is only a 0.01% chance that an F-values this large could occur due to noise. Adequate precision measures the signal to noise ratio and it was 35.917 for LP series foams and 22.452 for SP series foams which indicates an adequate signal. Developed models can be used to navigate the design space. The parity plots of the experimental results and modelled foaming start time of the developed rigid PUR foam are depicted in Figure S3 and they show an excellent fit of the developed model.

Table S4. ANOVA and the equation in terms of actual factors for the LP series and SP series rigid PUR foams foaming start time parameter response surface models and the R^2 values of the model.

Factors	Symbol	Foaming start time equation LP series	F-value	Sum of Squares	Foaming start time equation SP series	F-value	Sum of Squares
Model			99.23	3766.82		45.23	1482.71
Intercept		+140.748			+30.282		
LF polyol content	A	-0.205			+0.064		
Opteon™1100 content	B	-0.247			+0.433		
Water content	C	-30.027			+4.417		
Catalyst content	D	-41.083			-8.0900		
	A·B	+0.0075					
	B·C				-0.0667		
	C·D	+2.976					
	C^2	+5.098					
	D^2	+4.618					
R^2		0.968			0.895		
Adequate Precision		35.917			22.452		

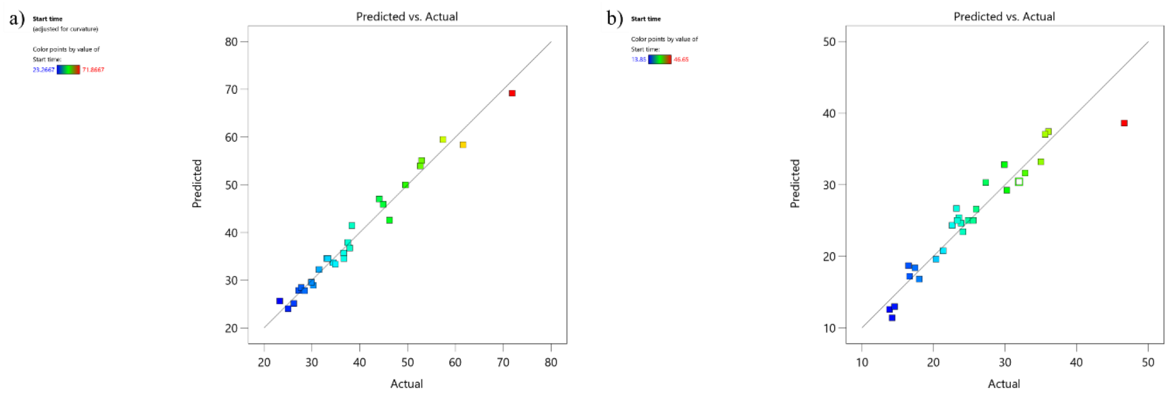

Figure S3. Parity plot of the foaming start time parameter response surface models: **a** – LP series foams; **b** –SP series foams.

Foam rise time MRSM

The LF polyol content, Opteon™1100 content, water content and catalyst content influence on the foaming rise time of developed two sets of rigid PUR foams was approximated by quadratic and 2FI models respectively and the ANOVA of the models is summarised in Table S5. The Model F-value of 225.40 and 29.63 for LP series foams and SP series foams respectively and it implies that the models are significant and there is only a 0.01% chance that an F-values this large could occur due to noise. Adequate precision measures the signal to noise ratio and it was 51.945 and 19.327 for LP series foams and SP series foams respectively which indicates an adequate signal. Developed models can be used to navigate the design space. The parity plots of the experimental results and modelled foaming rise time of the developed rigid PUR foam are depicted in Figure S4 and they show an excellent fit of the developed model.

Table S5. ANOVA and the equation in terms of actual factors for the LP series and SP series rigid PUR foams rise time parameter response surface models and the R^2 values of the model.

**Factors**	**Symbol**	**Rise time equation LP series**	**F-value**	**Sum of Squares**	**Rise time equation SP series**	**F-value**	**Sum of Squares**
Model			225.40	$1.79 \cdot 10^5$		29.63	13830.08
Intercept		+530.061			+93.470		
LF polyol content	A	-0.289			+0.165		
Opteon™1100 content	B	+10.156			+2.243		
Water content	C	-30.076			+20.144		
Catalyst content	D	-188.216			-23.933		
	B·C				-0.667		
	B·D	-2.033					
	D^2	+26.266					
R^2		0.981			0.846		
Adequate Precision		51.945			19.327		

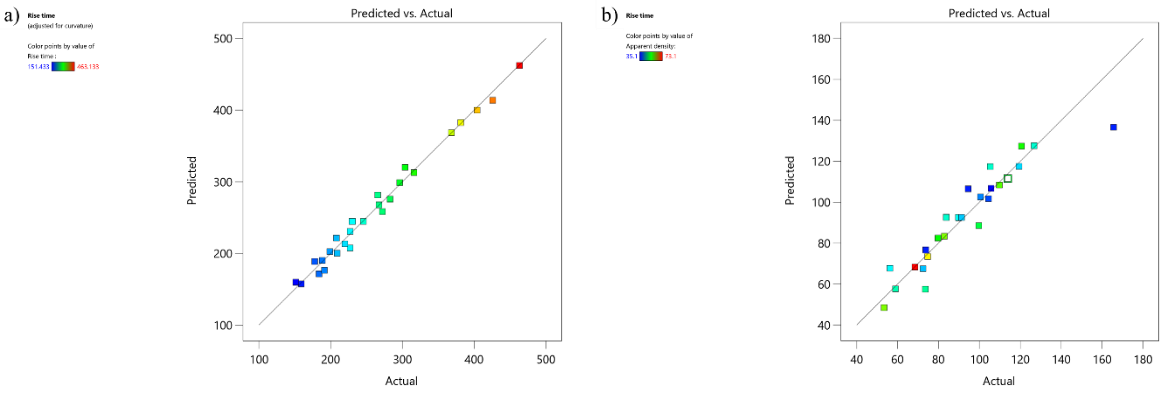

a b

Figure S4. Parity plot of the foam rise time parameter response surface models: **a** – LP series; **b** –SP series.

Shrinkage after 24 h MRSM

The LF polyol content, Opteon™1100 content, water content and catalyst content influence on the initial shrinkage of the developed two series of rigid PUR foams was approximated by a linear models and the ANOVA of the models is summarised in Table S6. The Model F-value was 16.48 and 1.45 for SP series foams and SP series foams, respectively. In the case of the LP series rigid PUR foams, the Model F-value implies that it is significant and there is only a 0.01% chance that an F-values this large could occur due to noise. However, in the case of the SP series rigid PUR foams, the model F-value is only 1.45, which implies that the model is not significant relative to the noise. Adequate precision measures the signal to noise ratio and it was 14.001 and 4.286 for LP series foams and SP series foams, respectively, which indicates an adequate signal. Developed models can be used to navigate the design space. However, the shrinkage data of the SP series rigid PU foams has to be critically evaluated as the F-value and R^2 is too low. The parity plots of the experimental results and modelled foam shrinkage after 24 h of the developed rigid PUR foam are depicted in Figure S5 and they show a fit of the developed model.

Table S6. ANOVA and the equation in terms of actual factors for the LP series and SP series rigid PU foams shrinkage after 24 h response surface models and the R^2 values of the model.

**Factors**	**Symbol**	**Shrinkage equation LP series**	**F-value**	**Sum of Squares**	**Shrinkage equation SP series**	**F-value**	**Sum of Squares**
Model			16.48	11.51		1.45	0.758
Intercept		-1.564			+0.889		
LF polyol content	A	+0.0234			+0.00736		
Opteon™1100 content	B	+0.0388			+0.0113		
Water content	C	+0.744			-0.0333		
Catalyst content	D	+0.0650			+0.0267		
R^2		0.704			0.0648		
Adequate Precision		14.001			4.286		

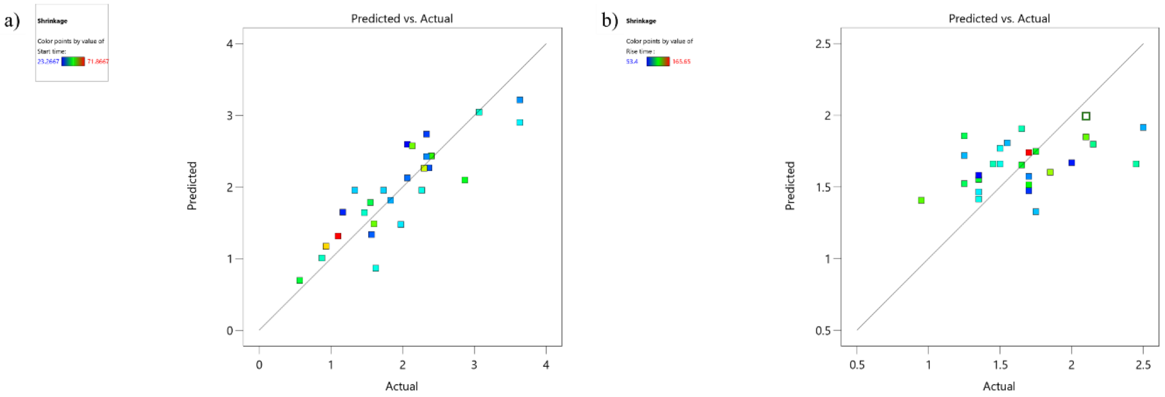

Figure S5. Parity plot of the foam shrinkage after 24 h parameter response surface models: **a** – LP series; **b** –SP series.

FTIR of LPO and SPO foams

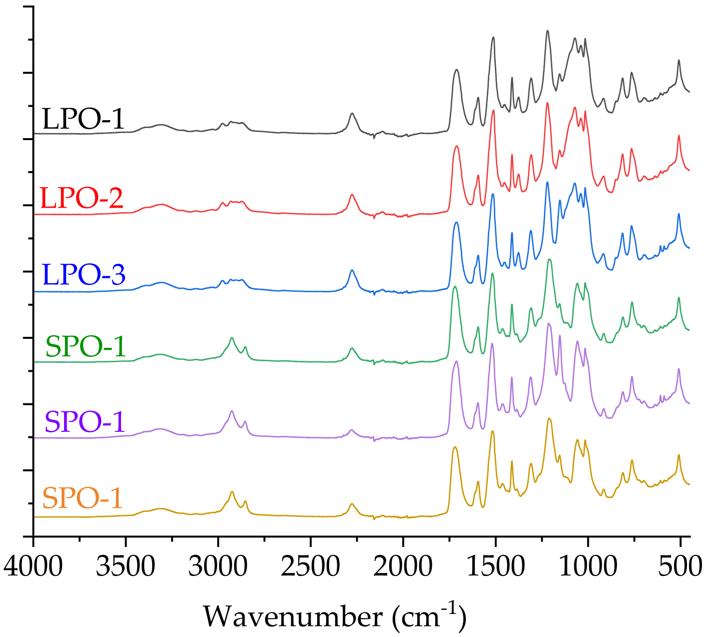

Figure S6. FTIR curves of LPO and SPO foams.

Optical microscopy images

Figure S4 displays previews of optical microscopy images of SPO and LPO foams, captured in both parallel (||) and perpendicular (⊥) directions to the foaming directions.

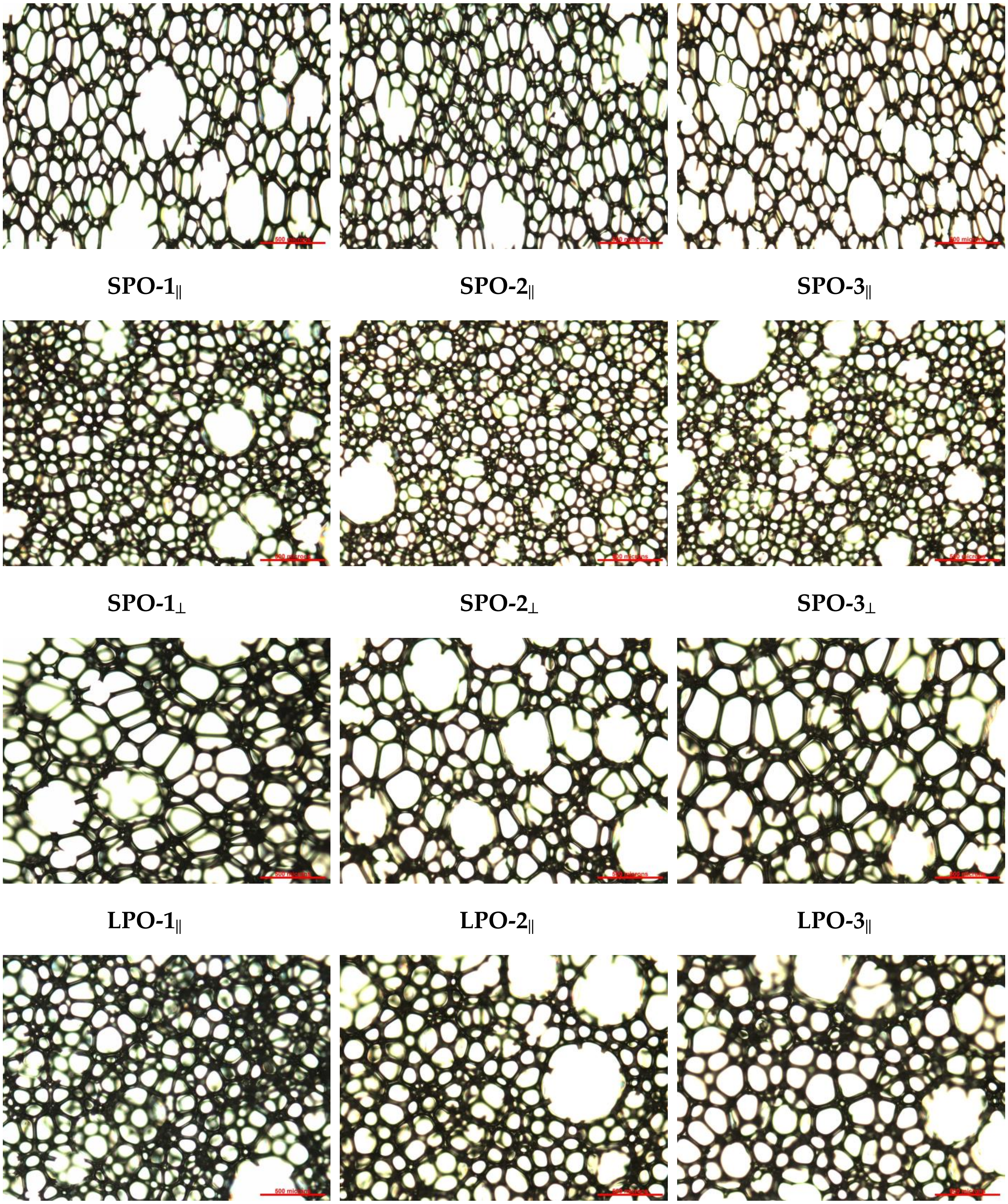

SPO-1$_{\parallel}$ **SPO-2$_{\parallel}$** **SPO-3$_{\parallel}$**

SPO-1$_{\perp}$ **SPO-2$_{\perp}$** **SPO-3$_{\perp}$**

LPO-1$_{\parallel}$ **LPO-2$_{\parallel}$** **LPO-3$_{\parallel}$**

LPO-1$_{\perp}$ **LPO-2$_{\perp}$** **LPO-3$_{\perp}$**

Figure S7. Optical microscopy images of SPO and LPO foams, captured in both parallel (||) and perpendicular (⊥) directions to the foaming directions.
